# Supplementary material for: Displacive Jahn–Teller Transition in NaNiO2
Source: J Am Chem Soc. 2024 Oct 14;146(43):29560–74. doi: 10.1021/jacs.4c09922 (PMC11528442; doi:10.1021/jacs.4c09922)
Supplement: Supplementary file 1 — ja4c09922_si_001.pdf [file ja4c09922_si_001.pdf]

Displacive Jahn–Teller transition in $NaNiO_2$: Supplementary Information

Liam A. V. Nagle-Cocco[a,*,†] Annalena R. Genreith-Schriever,[‡] James M. A. Steele,[†,‡] Camilla Tacconis,[†] Joshua D. Bocarsly[b,†,‡] Olivier Mathon,[¶] Joerg C. Neuefeind,[§] Jue Liu,[§] Christopher A. O'Keefe,[‡] Andrew L. Goodwin,[‖] Clare P. Grey,[‡] John S. O. Evans,[⊥] and Siân E. Dutton[c*,†]

†*Cavendish Laboratory, University of Cambridge, JJ Thomson Avenue, Cambridge, CB3 0HE, United Kingdom.*

‡*Yusuf Hamied Department of Chemistry, University of Cambridge, Cambridge, CB2 1EW, United Kingdom.*

¶*European Synchrotron Radiation Facility, 38043 Grenoble Cedex 9, France.*

§*Spallation Neutron Source, Oak Ridge National Laboratory, Oak Ridge, TN 37831, United States of America.*

‖*Inorganic Chemistry Laboratory, Department of Chemistry, University of Oxford, Oxford, OX1 3QR, United Kingdom.*

⊥*Department of Chemistry, Durham University, Durham, DH1 3LE, United Kingdom.*

E-mail: lavn2@cam.ac.uk; sed33@cam.ac.uk

[a]Email: lavn2@cam.ac.uk.
[b]Present address: Department of Chemistry, University of Houston, Texas, United States of America.
[c]Email: sed33@cam.ac.uk.

Contents

List of Figures 4

List of Tables 7

S1 Q_2 and Q_3 van Vleck modes 9

S1.1 Derivation of Q_3 as used in the calculations of Rietveld-refined structures . . 9

S2 Variable-temperature X-ray diffraction results 11

S2.1 Q_3 with heating 12

S2.2 Conductivity data against phase fraction 14

S3 Pair Distribution Function: analysis details 15

S3.1 Small box PDF analysis 15

S3.2 Generating the $NaNiO_2$ cell in orthorhombic setting 17

S3.3 Ni-O and Na-O bond distributions 17

S3.4 Temperature-dependence of bond length distortion index of NaO_6 octahedra 20

S3.5 Temperature-dependence of the $E_g(Q_2, Q_3)$ van Vleck modes from PDF data 21

S3.6 Ni-Ni atomic distance distributions 22

S3.7 Testing an orbitally-disordered starting configuration at 500 K 25

S3.7.1 Generating the orbitally-disordered starting configuration 26

S3.7.2 Results from big box fitting from JT-distorted starting configuration 31

S3.8 Visualising the Ni-O bond lengths from big box neutron PDF analysis in terms of short and long bonds 36

S3.9 Big box analysis without BVS restraints 38

S3.10Trans-octahedral O-O distances from big box PDF analysis 42

S3.11Repeating refinements at 500 K for repeatability 43

S4 X-ray absorption spectroscopy: additional figures 44

S4.1 Derivative of μ with energy 44

S4.2 EXAFS fitting 44

S5 *Ab initio* molecular dynamics: additional plots 47

S5.1 $E_g(Q_2, Q_3)$ van Vleck plots 47

S5.2 Convoluting histograms of bond lengths with Q_{max} 50

S6 [23] Nuclear Magnetic Resonance figure 52

S7 Tabulated data 53

S7.1 Big box neutron PDF: lattice parameters of supercell 53

S7.2 Big box neutron PDF: octahedral properties for supercell refined against experimental data 55

S7.3 Big box neutron PDF: octahedral properties for supercell refined against penalties only 58

S7.4 EXAFS data: Figures of merit 61

S7.5 EXAFS data: Refined parameters 64

S7.6 EXAFS data: Q_3 parameter 72

References 74

List of Figures

S1 Q_2 and Q_3 van Vleck modes 9

S2 Example Rietveld refinements from the variable-temperature synchrotron diffraction. 11

S3 R_{wp} with temperature for the synchrotron Rietveld refinements. 12

S4 Octahedral volume with temperature based on Rietveld refinement of synchrotron data 13

S5 Q_3 with temperature for the monoclinic $NaNiO_2$ phase, from synchrotron diffraction data 13

S6 Conductivity of $NaNiO_2$ on heating, compared with rhombohedral phase fraction 14

S7 Small box fits to the neutron PDF data 16

S8 Ni-O and Na-O bond length distributions from big box neutron PDF analysis. 18

S9 Standard deviation versus mean Na-O and Ni-O bond lengths from big box neutron PDF analysis. 19

S10 Temperature-dependence of bond length distortion index of NaO_6 octahedra. 20

S11 Van Vleck $E_g(Q_2, Q_3)$ plots for the big box neutron PDF analysis. 21

S12 Histogram of E_g distortion magnitude from big box neutron PDF analysis. . 21

S13 The Ni-Ni interatomic distance distribution at 293 K from big box neutron PDF analysis. 22

S14 The Ni-Ni interatomic distance distribution at 450 K from big box neutron PDF analysis. 23

S15 The Ni-Ni interatomic distance distribution at 500 K from big box neutron PDF analysis. 24

S16 Example O-Ni-O layers, and energy dependence on cycle, for generating locally JT-distorted $2 \times 2 \times 1$ supercells, with $T = 0.5\,T_c$. 28

S17 Example O-Ni-O layers, and energy dependence on cycle, for generating locally JT-distorted $10 \times 6 \times 1$ supercells, with $T = 0.5\,T_c$. 29

S18 Example O-Ni-O layers, and energy dependence on cycle, for generating locally JT-distorted $10 \times 6 \times 1$ supercells, with $T = 50\,T_c$. 30
S19 Ni-O bond length distributions for big box analysis of 500 K neutron PDF data, using starting configurations with random Jahn–Teller distortions, with BVS restraints enabled. 33
S20 Ni-O bond length distributions for big box analysis of 500 K neutron PDF data, using starting configurations with random Jahn–Teller distortions, with BVS restraints enabled. 34
S21 Probability distribution of ρ_0 for the big box PDF analysis from Monte Carlo-generated starting structures. 35
S22 Histogram of Ni-O bond length from big box neutron PDF analysis, presented as the average of the 4 smallest and 2 longest bonds per octahedron. 37
S23 Histograms of bond length from big box neutron PDF analysis, where BVS restraints were not applied. 39
S24 Bragg and PDF neutron diffraction data, fit via a big box method where BVS restraints were not applied. 40
S25 A histogram of the distribution of calculated NiO_6 Q_3 values, obtained via big box analysis where BVS restraints were not applied. 40
S26 A histogram of the probability distribution of ρ_0 obtained via big box analysis where BVS restraints were not applied. 41
S27 Histograms of O-O bond length from big box neutron PDF analysis. 42
S28 Probability distributions $P(\rho_0)$ for several repeats of the 500 K big box PDF analysis without JT-distorted starting configurations. 43
S29 $d\mu/dE$ for the X-ray absorption data around the Ni K edge, with temperature. 44
S30 Figures of merit for the 5 different models fit to the EXAFS data. 45
S31 Ni-Ni interatomic distances obtained from fitting the JT-distorted {2,2} model to EXAFS data during heating. 45

S32 Example EXAFS fits using JT-distorted {2,0} and JT-undistorted {1,0} models at the Ni-O peak. 46
S33 Ni-O bond lengths from EXAFS using JT-distorted {2,0} model. 46
S34 Van Vleck $E_g(Q_2, Q_3)$ plots for the *ab initio* molecular dynamics simulations during heating. 48
S35 Van Vleck $E_g(Q_2, Q_3)$ plots for the *ab initio* molecular dynamics simulations during cooling. 49
S36 Histograms of Ni-O bond lengths from AIMD convolved with experimental Q_{max} 51
S37 Static and dynamic ^{23}Na nuclear magnetic resonance 52

List of Tables

S1 Fitting metrics R_{wp} for small box analysis of neutron PDF data 15

S2 Outputs from big box analysis of neutron pair distribution function when varying the starting configuration using a Monte Carlo approach. 32

S3 Comparison of R_{wp} from fitting the big box model to the neutron PDF and Bragg data, with and without bond valence sum restraints. 39

S4 Lattice parameters of supercell used for big box neutron PDF analysis. . . . 54

S5 NiO_6 octahedral properties in the supercell obtained by big box refinement against neutron PDF data 56

S6 NaO_6 octahedral properties in the supercell obtained by big box refinement against neutron PDF data 57

S7 NiO_6 octahedral properties in the supercell obtained by big box refinement against penalties 59

S8 NaO_6 octahedral properties in the supercell obtained by big box refinement against penalties 60

S9 Figures of merit obtained by fitting various models to the EXAFS data as a function of temperature for the Ni-O shell only. 62

S10 Figures of merit obtained by fitting various models to the EXAFS data as a function of temperature for the Ni-O and Ni-Ni shells. 63

S11 Refined parameters for all paths from fitting a JT-undistorted {1,0} model to the EXAFS data for the Ni-O shell only. 65

S12 Refined parameters for all paths from fitting a JT-distorted {2,0} model to the EXAFS data for the Ni-O shell only. 66

S13 Refined parameters for all paths from fitting a {1,1} model to the EXAFS data for the Ni-O and Ni-Ni shells. 67

S14 Refined parameters for all paths from fitting a {2,1} model to the EXAFS data for the Ni-O and Ni-Ni shells (1 of 2). 68

S15 Refined parameters for all paths from fitting a {2,1} model to the EXAFS data for the Ni-O and Ni-Ni shells (2 of 2). 69

S16 Refined parameters for all paths from fitting a {2,2} model to the EXAFS data for the Ni-O and Ni-Ni shells (1 of 2). 70

S17 Refined parameters for all paths from fitting a {2,2} model to the EXAFS data for the Ni-O and Ni-Ni shells (2 of 2). 71

S18 Refined Q_3 parameter from each model and set of shells used in fitting the EXAFS data. 73

S1 Q_2 and Q_3 van Vleck modes

In the paper, we refer to the Q_2 and Q_3 van Vleck modes.[1,2] Figure S1 shows a visual depiction of these two modes. Only the Q_3 mode is relevant to $NaNiO_2$, but Jahn–Teller distortions can manifest as a linear combination of both modes.

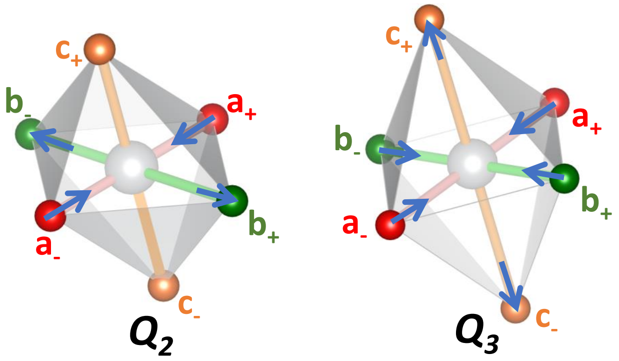

Figure S1: The Q_2 and Q_3 distortion modes, shown for an NiO_6 octahedron with atoms labelled a, b, or c according to the axis on which they are located, and with + or - depending on their position along that axis relative to the Ni atom. Adapted with permission of the International Union of Crystallography from Nagle-Cocco and Dutton (2024).[2]

S1.1 Derivation of Q_3 as used in the calculations of Rietveld-refined structures

Calculation of van Vleck Q_3 mode in Topas from Rietveld refinement is done with the assumption of no angular distortion introduced by Kanamori.[3] Although there is in fact angular distortion in the NiO_6 octahedra in $NaNiO_2$, it has been shown that this has no significance on the calculation of Q_3.[2] We here derive the form of Q_3 used to calculate Q_3 from the Rietveld refined structures, obtained from the sequential Rietveld refinement in Section S2.

For an octahedron with 3 different core-ligand bond lengths, and each bond being 180° around the core atom from its equally long counterpart, Kanamori approximated the $E_g(Q_2, Q_3)$ van Vleck modes[3] as follows:

$$Q_2 = r_3 - r_1 \tag{S1}$$

$$Q_3 = \frac{(2r_2 - r_1 - r_3)}{\sqrt{3}} \tag{S2}$$

where r_1, r_2, and r_3 are the three different core-ligand bond lengths. We note that we do not include the factors $2/\sqrt{2}$ from Kanamori's work;[3] we do this deliberately, to enable direct comparison with values obtained using VanVleckCalculator.[2,4] These equations have often been used in the literature for systems with non-zero Q_2 components.[5–28] Commonly, $r_1 = s$, $r_2 = m$, and $r_3 = l$ where s, m, and l are the small, medium, and long bond lengths in an octahedron.

For the NiO_6 octahedron in $NaNiO_2$, there is a purely tetragonal Q_3-type distortion, meaning that $s = m$. We define $r_3 = r_1 = s$ and $r_2 = l$. This is equivalent to performing a transformation equivalent to a rotation by 120° in the angle $\phi = \arctan\left(\frac{Q_2}{Q_3}\right)$ and set:

$$Q_2 = m - s = 0 \tag{S3}$$

$$Q_3 = \frac{(2l - m - s)}{\sqrt{3}} = \frac{2(l - s)}{\sqrt{3}} \tag{S4}$$

where we note that this leaves the magnitude $\rho_0 = \sqrt{Q_2^2 + Q_3^3}$ unchanged.

We therefore use Equation S4 to calculate Q_3 for the variable-temperature diffraction data, rather than Equation S2, so we can describe the distortion using a single parameter.

S2 Variable-temperature X-ray diffraction results

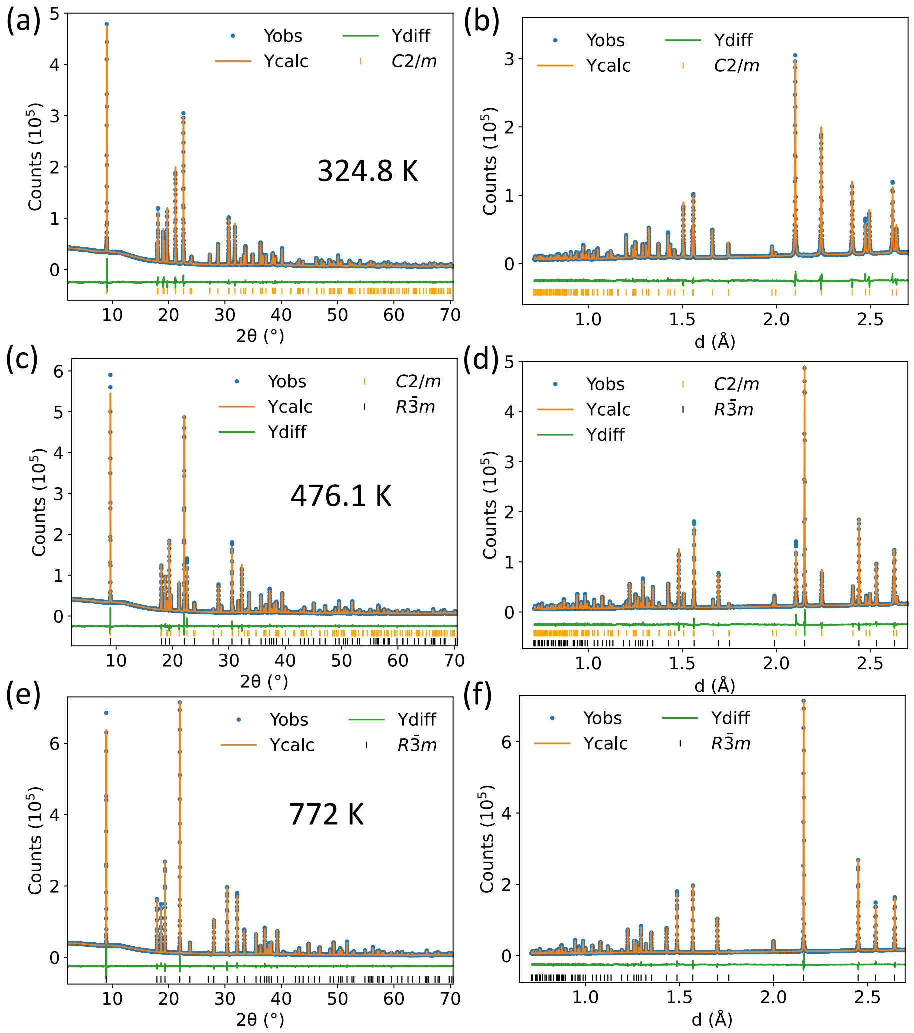

Figure S2: Example Rietveld refinements for the synchrotron diffraction, in the (a,b) monoclinic regime, (c,d) mixed-phase regime, and (e,f) rhombohedral regime. Data is presented in both 2θ ($\lambda = 0.824110$ Å) and d.

Figure S2 shows example Rietveld refinements in the mixed-phase and the two single-phase temperature regimes, from the variable-temperature diffraction experiment on I11. Figure S3 shows the R_{wp} of fits along with the rhombohedral phase fraction, calculated using the refined scale factors from Rietveld refinement. Figure S4 shows the octahedral volume of NiO_6 and NaO_6 octahedra with temperature.

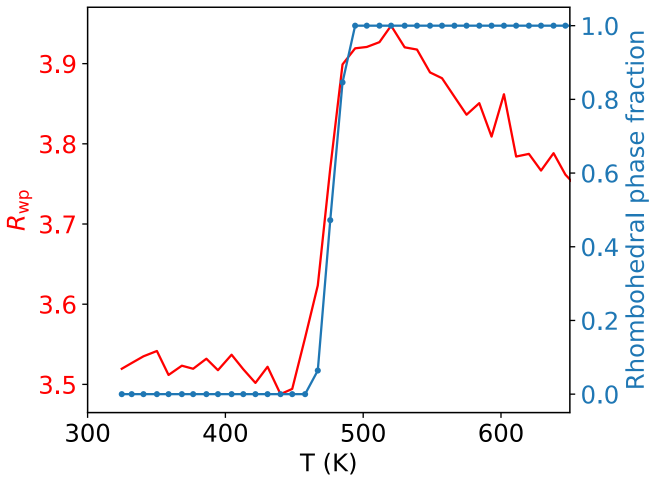

Figure S3: R_{wp} with temperature for the synchrotron Rietveld refinements, performed using a $C2/m$ space group for the monoclinic regime and a $R\bar{3}m$ space group for the rhombohedral regime, with both phases refined within the mixed-phase regime. For reference, the fraction of the sample in the rhombohedral phase is also superimposed to show an increase in R_{wp} with the transition, likely due to a reduction in the number of fitted parameters.

S2.1 Q_3 with heating

To understand the impact of heating on the Jahn–Teller distortion in the average structure, we use the van Vleck[1] Q_3 mode to quantify the tetragonal distortion of the NiO_6 octahedra, calculated using $Q_3 = \frac{2(l-s)}{\sqrt{3}}$ (derived in Section S1.1). Q_3 is zero within the rhombohedral phase, but has a finite value in the monoclinic phase, Figure S5.

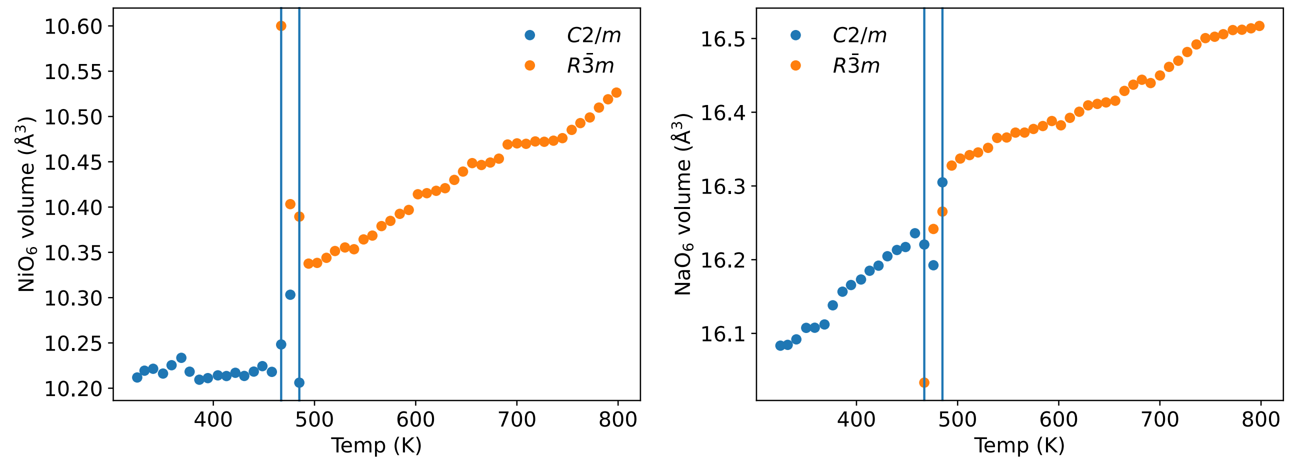

Figure S4: Octahedral volume with temperature based on Rietveld refinement of synchrotron data. The region between the vertical lines is the mixed-phase regime in which refined oxygen positions are unstable.

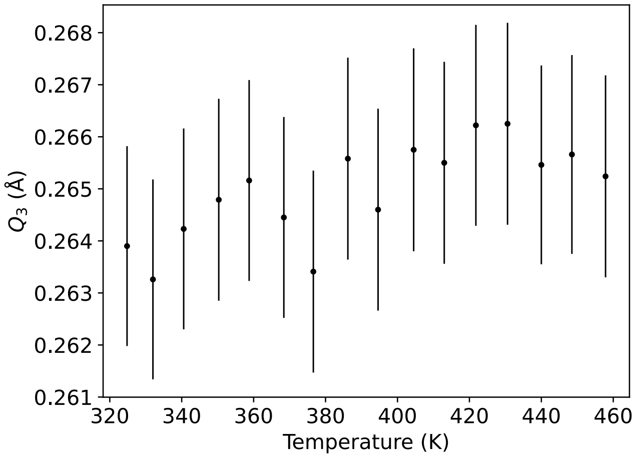

Figure S5: Q_3 parameter for the monoclinic phase as obtained by Rietveld refinement of the synchrotron diffraction data. For the rhombohedral phase, $Q_3 = 0$ by symmetry. For both phases, $Q_2 = 0$ by symmetry.

S2.2 Conductivity data against phase fraction

As mentioned in the main text, we present the conductivity data previously published by Delmas *et al.* (1994)[29] against the rhombohedral phase fraction in Figure S6.

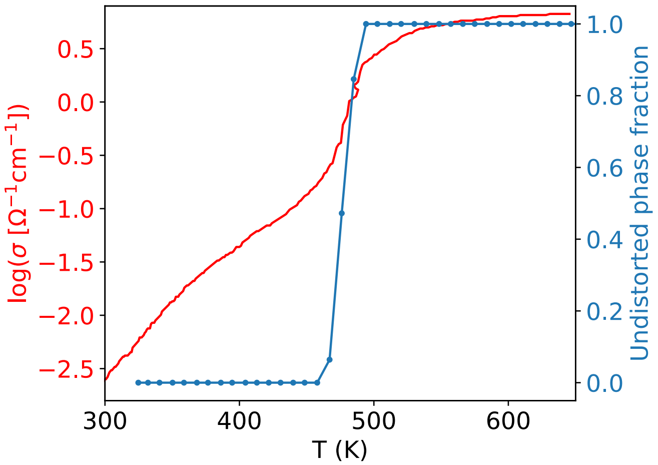

Figure S6: Conductivity data on $NaNiO_2$, taken from Delmas *et al.* (1994)[29] using DataThief-III.[30] Data is superimposed over the rhombohedral phase fraction obtained from scale factors Rietveld refined from the synchrotron diffraction data on heating presented in this study.

S3 Pair Distribution Function: analysis details

In this section, we present additional analysis from the neutron Pair Distribution Function (PDF) data, which was obtained from the NOMAD instrument[31] at Oak Ridge National Laboratory.

S3.1 Small box PDF analysis

Before attempting the big box PDF fitting described in the main text and subsequently in this SI, we attempted a small-box analysis of the neutron PDF data. Fitting was performed in the range 1 Å to 10 Å in real space, without using the reciprocal space data. Both datasets were fit with a monoclinic and rhombohedral cell, with the former able to accommodate a Jahn–Teller distortion and the latter unable to. The fits are shown in Figure S7.

At 293 K, the rhombohedral fit is very poor throughout the fitting range; at low-r the rhombohedral cell is unable to reproduce the bond length splitting of the Ni-O coordination cell, and at higher r the fit is poor because the layer glide associated with the monoclinic distortion is not reproduced. The monoclinic cell is able to fit the PDF data well, consistent with the reciprocal space diffraction data.

At 500 K, both the monoclinic and rhombohedral unit cells fit the data well. In the case of the monoclinic cell, the Ni-O bond lengths actually converge to be approximately equal: $4 \times (1.980 \pm 0.003)$ Å and $2 \times (2.021 \pm 0.005)$ Å. While this is not quite a convergence, such a change in Ni-O bond lengths would not be consistent with a local Jahn–Teller distortion.

Table S1 shows the fitting metrics from this small box fitting.

Table S1: Fitting metric R_{wp} for small box analysis of neutron PDF data.

T	$R\bar{3}m$	$C2/m$
293 K	64.780	9.994
500 K	8.835	8.789

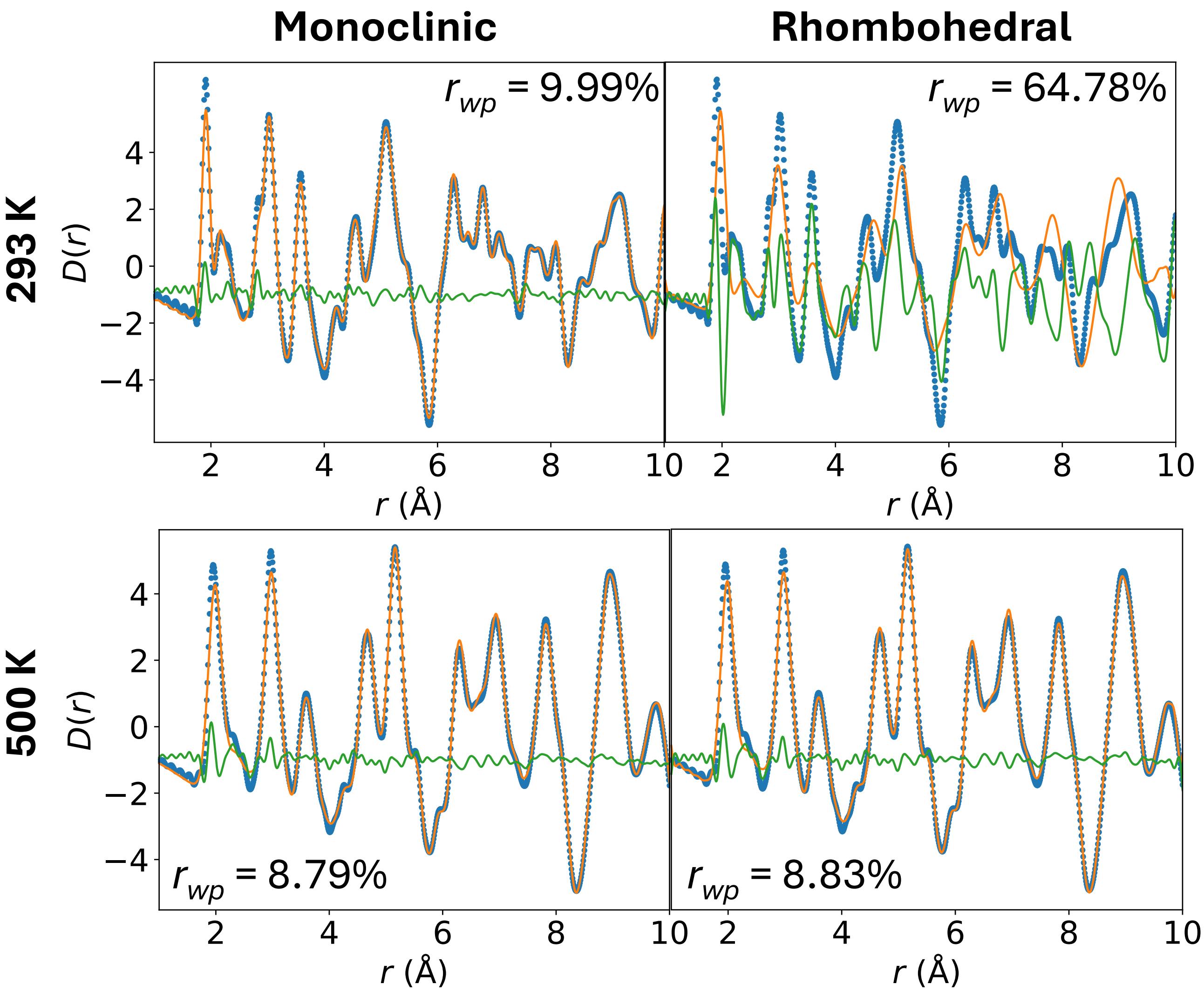

Figure S7: Small box fits, by real-space Rietveld refinement in Topas, of the neutron diffraction data at 293 K (top) and 500 K (bottom), using a monoclinic $C2/m$ JT-distorted structure (left) and a rhombohedral $R\bar{3}m$ JT-undistorted structure (right).

S3.2 Generating the $NaNiO_2$ cell in orthorhombic setting

For the big-box PDF analysis, it was desirable to use a supercell as close as possible to cubic. However, the high-symmetry phase commonly used for $NaNiO_2$ is rhombohedral ($R\bar{3}m$) with $\gamma = 120°$. We chose to transform this high-symmetry cell into a pseudo-orthorhombic unit cell, to use as the parent cell from which to generate the supercell. The transformation matrix used to obtain the unit cell with orthorhombic setting from the rhombohedral cell is as follows:

$$\begin{bmatrix} a_o \\ b_o \\ c_o \end{bmatrix} = \begin{bmatrix} 1 & 1 & 0 \\ 1 & -1 & 0 \\ 0 & 0 & -1 \end{bmatrix} \begin{bmatrix} a_r \\ b_r \\ c_r \end{bmatrix} \tag{S5}$$

where subscripts o and r denote the orthorhombic and rhombohedral lattice parameters respectively.

S3.3 Ni-O and Na-O bond distributions

Figure S8 shows the distribution of the smallest to largest M-O (M=Na,Ni) bond length in each octahedron from the output of big box PDF analysis. As discussed in the main text, there is a splitting into 4+2 bond lengths below 500 K for the NiO_6 octahedra consistent with tetragonally-elongated octahedra, indicating a Jahn–Teller effect.

Figure S9(a,b) shows the standard deviation against the mean bond length, for each Ni-O and Na-O bond in an octahedron in ascending order of size. It can be seen that, typically, the bond lengths increase with heating, but for the Ni-O distributions the mean 5th and 6th smallest bonds actually decrease in mean length at 500 K compared with the lower temperatures. This is consistent with the loss of the Jahn–Teller distortion. We also see that NaO_6 octahedra exhibit a consistent dependence of standard deviation on size ranking within the octahedron; standard deviation is smallest for the intermediate bond lengths, largest for the longest bond lengths, and the smallest bond lengths have medium standard

deviations. This is the same for NiO_6 octahedra at 500 K which are Jahn–Teller-undistorted, but the JT-distorted NiO_6 octahedra at 293 K and 450 K exhibit a different dependence. This is likely not a real physical effect but rather the consequence of large tails in the low-r region which get fit by small Ni-O bonds. With BVS restraints removed, as in Figure S9(c,d), the issue is even more severe consistent with the general findings discussed later in Section S3.9.

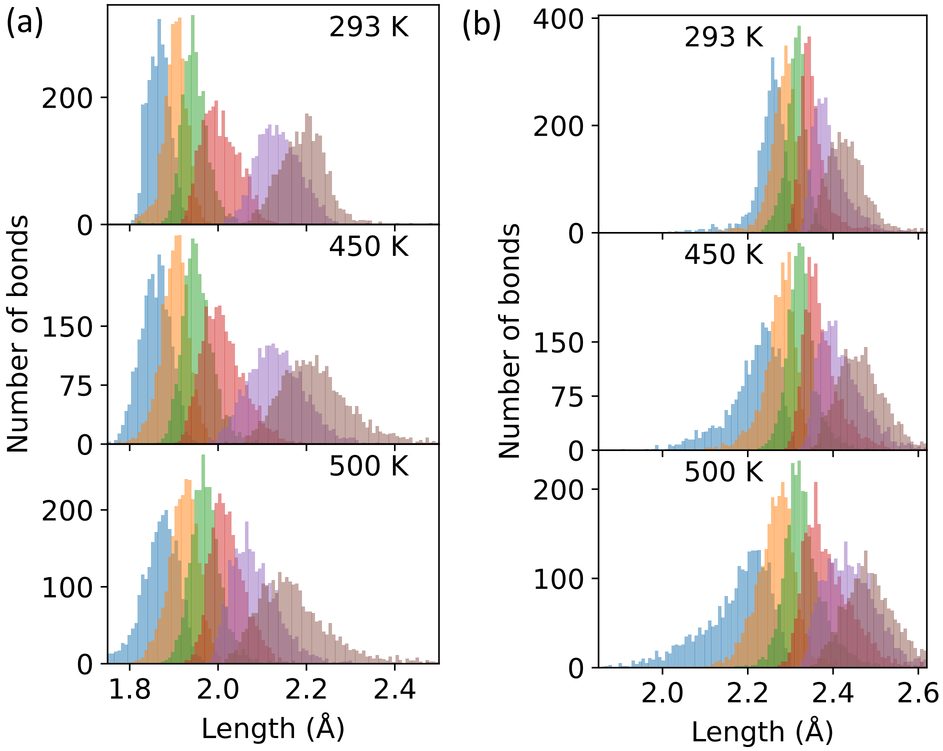

Figure S8: The (a,c) Ni-O and (b,d) Na-O bond length distributions at 293 K, 450 K, and 500 K from big box neutron PDF analysis both (a,b) with and (c,d) without BVS-based restraints.

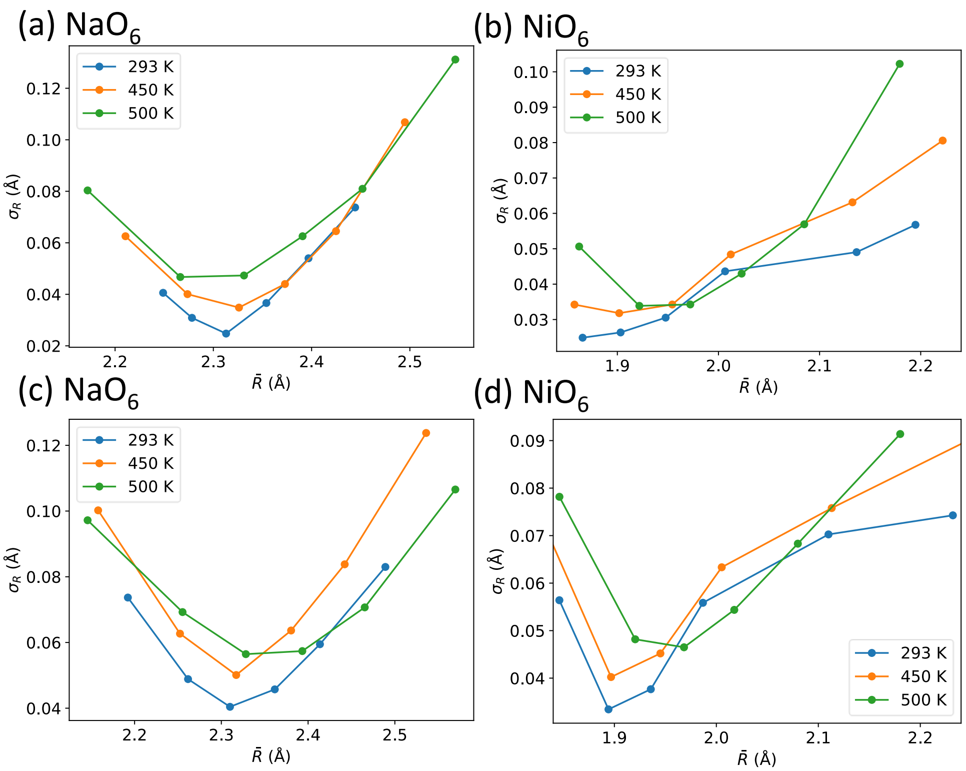

Figure S9: The (a,c) Na-O and (b,d) Ni-O bond distributions, represented as a standard deviation of each set of bonds shown in Figure S8 against the mean. (a,b) are calculated with BVS restraints and (c,d) are calculated without.

S3.4 Temperature-dependence of bond length distortion index of NaO_6 octahedra

Figure S10 shows the temperature-dependence of the bond length distortion index of NaO_6 octahedra from big box PDF analysis. It can be seen that the distribution and mean bond length distortion index increases with heating.

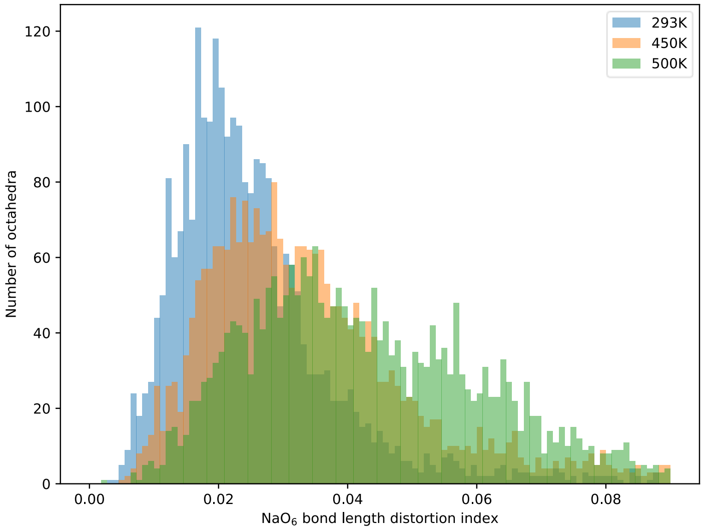

Figure S10: Histograms of bond length distortion index of NaO_6 octahedra from big box neutron PDF analysis.

S3.5 Temperature-dependence of the $E_g(Q_2, Q_3)$ van Vleck modes from PDF data

Figure S11 shows heat maps where intensity is proportional to the number of octahedra at each $E_g(Q_2, Q_3)$ point from big box neutron PDF analysis. Figure S12 shows a histogram of the magnitude $\rho_0 = \sqrt{Q_2^2 + Q_3^2}$ for all octahedra in the same supercells. We can see disappearance of the preference for Q_3 distortion and reduction in the magnitude overall.

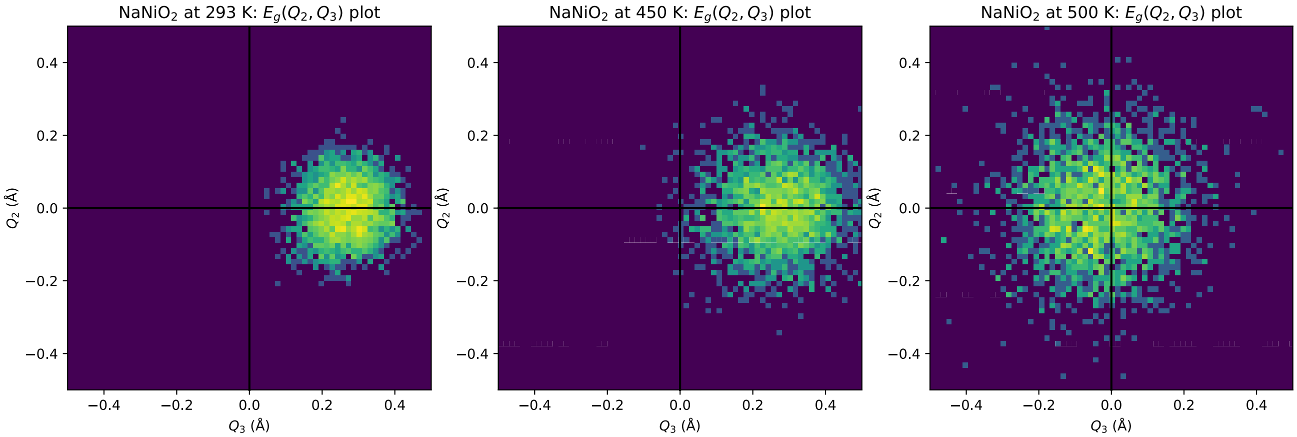

Figure S11: Van Vleck $E_g(Q_2, Q_3)$ plots for the big box neutron PDF analysis. These 2D histograms show the calculated modes for all octahedra in the supercell, as calculated using VanVleckCalculator.[2]

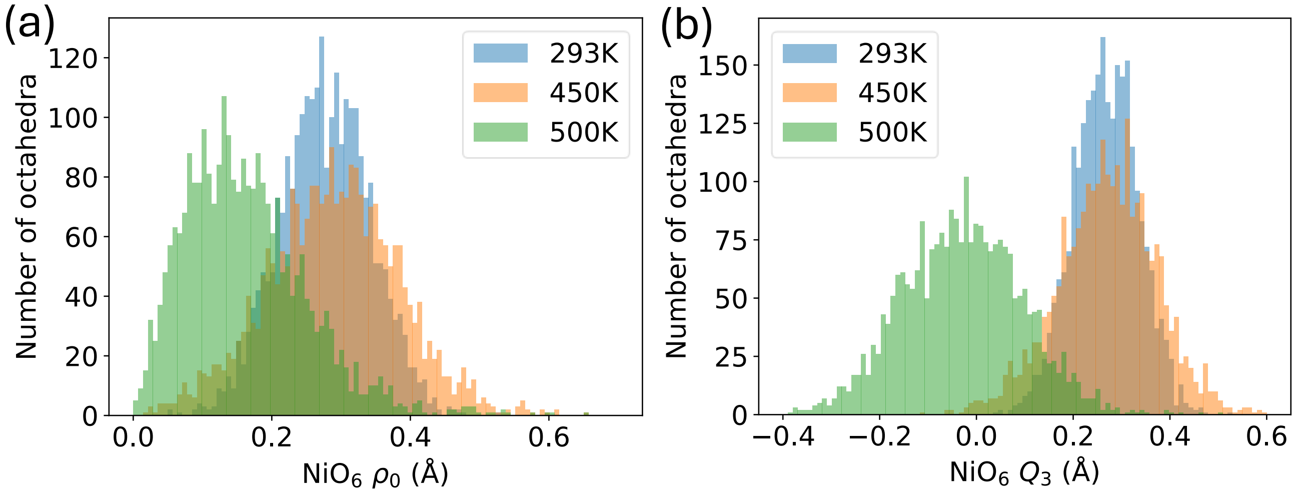

Figure S12: Histograms of (a) E_g distortion magnitude, $\rho_0 = \sqrt{Q_2^2 + Q_3^2}$, and (b) Q_3 from big box neutron PDF analysis as calculated using VanVleckCalculator.[2]

S3.6 Ni-Ni atomic distance distributions

Ni-Ni interatomic distances from the supercells obtained by big box PDF analysis are plotted in Figures S13, S14, and S15.

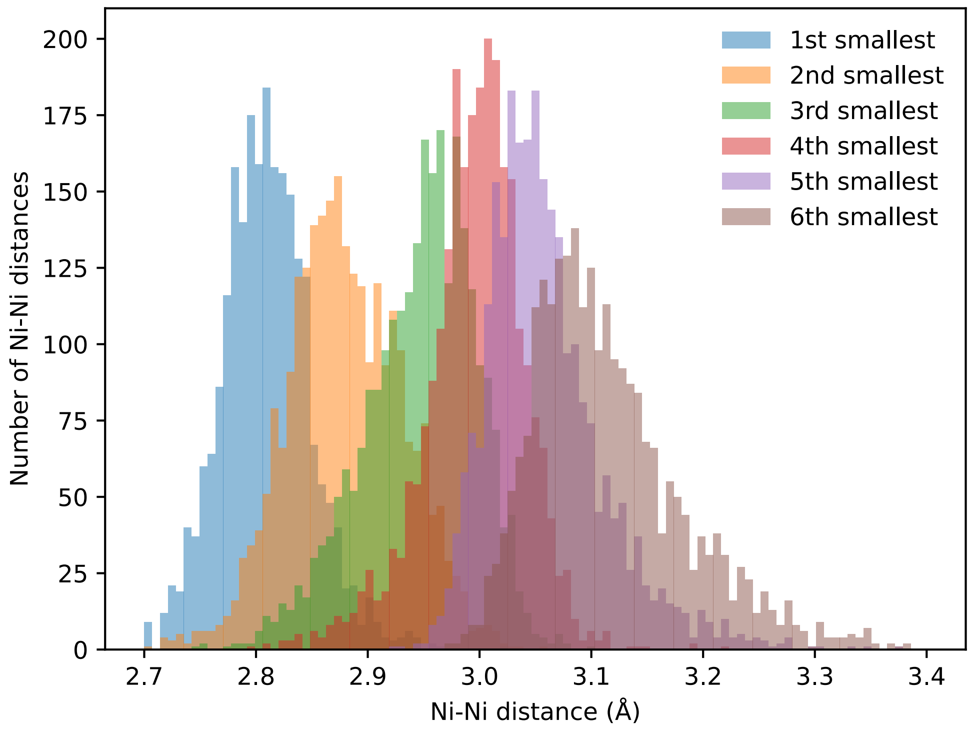

Figure S13: The Ni-Ni interatomic distance distribution at 293 K, as obtained from the big box analysis of the neutron Pair Distribution Function data. The smallest to 6th smallest interatomic distance is for the six Ni-Ni distances of each Ni cation.

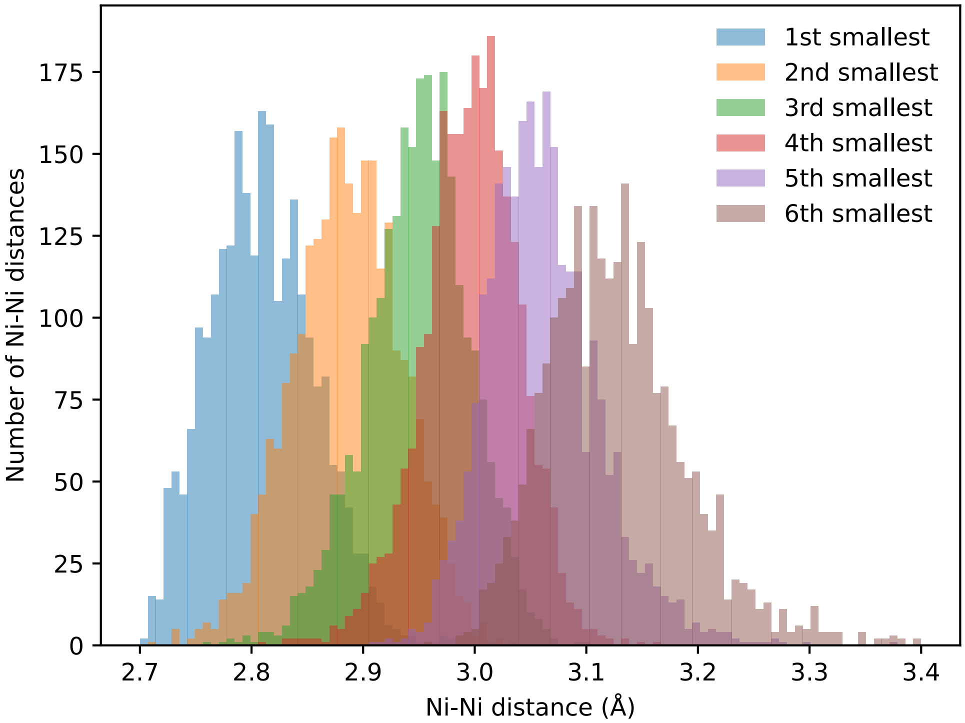

Figure S14: The Ni-Ni interatomic distance distribution at 450 K, as obtained from the big box analysis of the neutron Pair Distribution Function data. The smallest to 6th smallest interatomic distance is for the six Ni-Ni distances of each Ni cation.

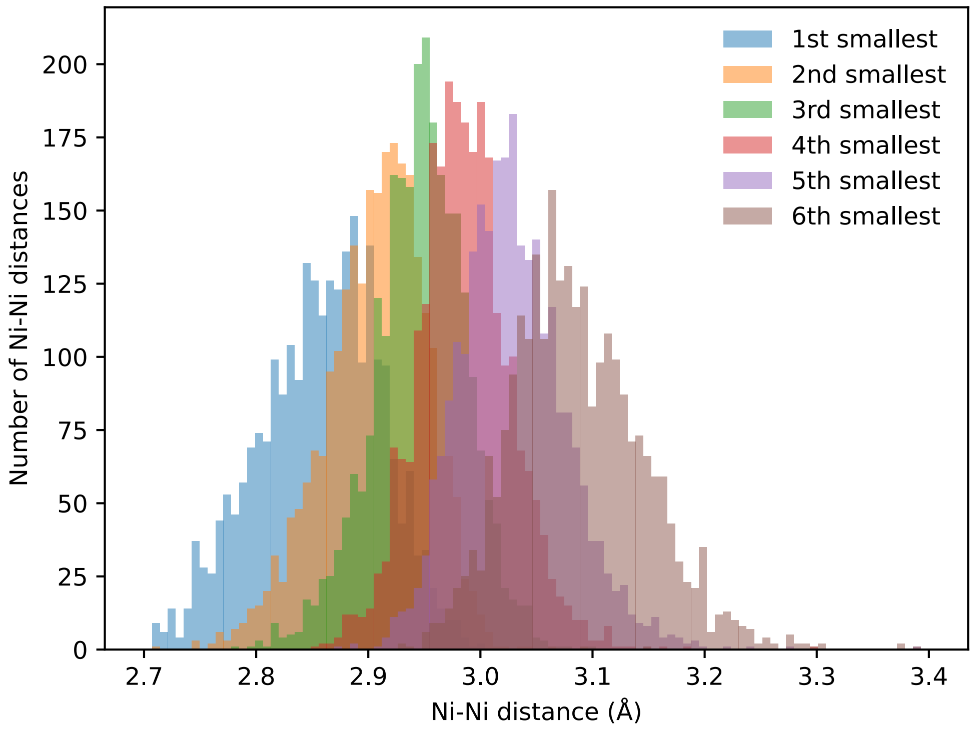

Figure S15: The Ni-Ni interatomic distance distribution at 500 K, as obtained from the big box analysis of the neutron Pair Distribution Function data. The smallest to 6th smallest interatomic distance is for the six Ni-Ni distances of each Ni cation.

S3.7 Testing an orbitally-disordered starting configuration at 500 K

The big box analysis of neutron PDF data presented in this study shows the absence of Jahn–Teller distortions at 500 K. However, the starting configuration of this analysis is JT-undistorted. Here, we present the result of similar big box analysis of neutron PDF data using starting $16 \times 9 \times 3$ supercells with starting configurations of disordered, non-cooperative JT-distorted octahedra. This starting configuration was generated using a Monte-Carlo technique, and then refined against the data following the same big box approach and set of restraints as was otherwise used. The results obtained were qualitatively identical to the results obtained from normal big box refinement, without biasing in favour of orbital disorder. This supports our conclusion that there is a displacive transition and the absence of Jahn–Teller distortion at 500 K in $NaNiO_2$.

S3.7.1 Generating the orbitally-disordered starting configuration

First, a pseudo-orthorhombic unit cell was obtained from the rhombohedral cell using the transformation in Equation S5. This was done in VESTA-3.[32] All subsequent steps were performed using custom-written Python 3[33] code, with PYMATGEN[34] used for handling crystal structures.

A Jahn–Teller-free supercell was then generated from the pseudo-orthorhombic cell. A global "energy" function was defined as follows:

$$E_{\text{total}} = \sum_i \text{abs}(n - 1) \tag{S6}$$

where the sum is performed over all oxygen sites, abs is a function that returns the absolute (non-negative) value of an input, and n is defined as the number of Jahn–Teller axes pointing at the given oxygen. This is the energy which, by a Monte Carlo approach, is minimised to induce orbital disorder. Note that a globally-defined, temperature parameter T is also defined, for use in Equation S7 defined later. T is defined in units of T_c, the ordering temperature. $T < 1$ would indicate a temperature below the ordering temperature, and $T >> 1$ would indicate a temperature above the ordering temperature.

The code then runs through a large number of cycles, where, during each cycle, the code will do the following (in this order):

1. Calculate total energy of the system using Equation S6
2. Select a random Ni site. If it is Jahn–Teller-distorted, remove the JT distortion.
3. Add a JT distortion to a random axis of the NiO_6 octahedron.
4. If the change decreases energy or leaves it unchanged, accept the change and skip to step 6.

5. If the change increases energy, accept it with a probability, P given by:

$$P = \exp\left[\frac{-\Delta E}{2T}\right] \tag{S7}$$

where the factor of 1/2 is present because ΔE is typically an integer multiple of 2.

6. Calculate total energy of the system using Equation S6. If total energy is not zero, return to step 1.

This is then run for a very large number of cycles with $T < T_c$ (specifically $T = 0.5\,T_c$) in order to obtain a crystal structure with local NiO_6 distortions, to see whether a locally-distorted, noncooperative JT distortion could be "frozen in" to then test as a starting configuration for big box refinement, analogous to the Potts model in $LaMnO_3$.[35] Experimentation with smaller supercells (as compared with the $14\times9\times3$ supercell used elsewhere in the study) was able to yield $E_{\text{total}} = 0$; this was only possible when there was a 1D chain of NiO_6 octahedra with parallel axes of elongation. For larger supercells, it was not feasible on reasonable timescales to obtain $E_{\text{total}} = 0$, and so Monte Carlo programmes were run for as long as reasonable before the resulting low-E cell was taken and used as the starting configuration for big box neutron PDF fitting. Figures S16(a,b) show examples of an O-Ni-O layer in a small supercell ($2 \times 2 \times 1$) which converged to $E_{\text{total}} = 0$. Figure S16(c) shows a similar configuration from a separate run on an identically-sized cell which actually recovered the room-temperature orbital ordering in $NaNiO_2$. Figure S16(d) shows the energy (as defined in Equation S6) versus number of cycles for the $2 \times 2 \times 1$ supercell in Figures S16(a,b). Figure S17(a,b,c) show equivalent O-Ni-O layers in a substantially larger supercell ($10 \times 6 \times 1$) which was not able to converge (starting $E_{\text{total}} = 718$; final $E_{\text{total}} = 200$). Here, we can see that the inability to converge is caused by domain walls between small nanodomains containing a cooperative JT distortion. We subsequently experimented with simulated annealing to further reduce the final energy, and in this fashion we were able to final $E_{\text{total}} = 24$, but further reducing energy just increases the size of the nanodomains as the energy is caused

by domain boundaries. We do not document the simulated annealing work here.

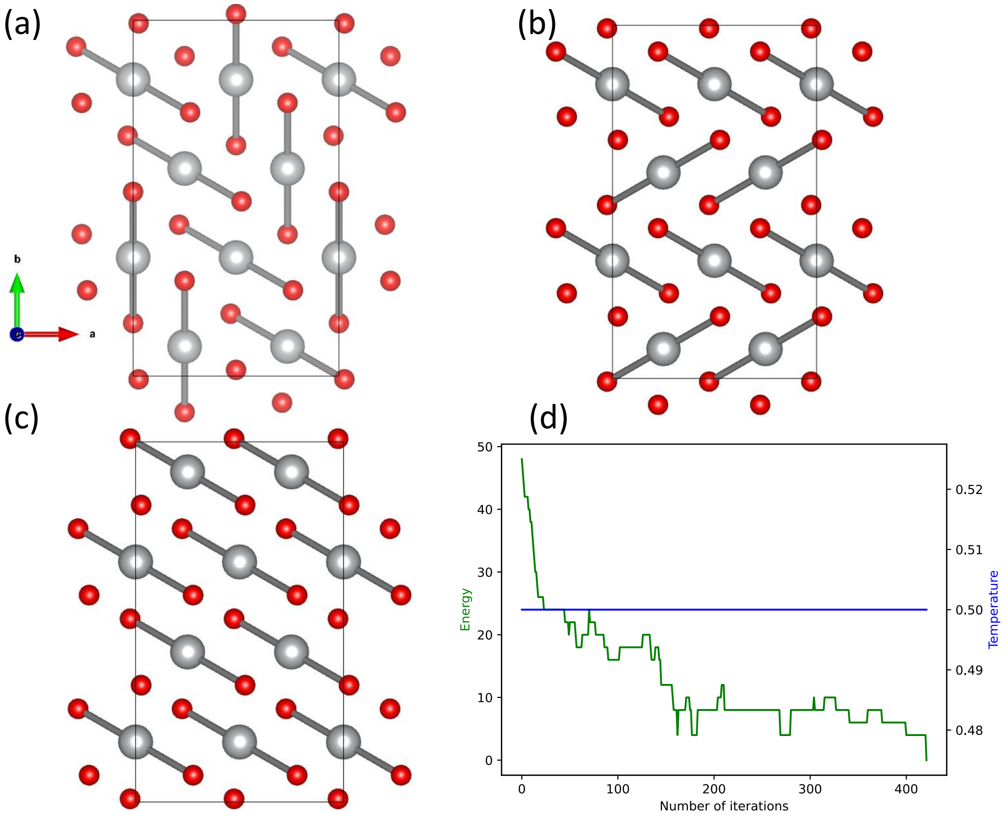

Figure S16: (a,b,c) Example O-Ni-O layers, and (d) energy dependence on cycle, for generating locally JT-distorted $2 \times 2 \times 1$ supercells. In this case, it was possible to reach $E_{\text{total}} = 0$ (as defined in Equation S6), but in the process 1D chains of NiO_6 octahedra with parallel axes of elongation were obtained. (a) and (b) were from the same Monte Carlo run described in (d), whereas (c) was from a different run. Grey atoms are Ni and red atoms are O. Only JT-elongated Ni-O bonds are shown.

We then ran the Monte Carlo calculation for 5,000 cycles with $T = 50\,T_c$, with the results shown in Figure S18. Using such high temperatures typically avoided the formation of domains, and leads to local ordering including many of those proposed by Chung *et al.*[36] in their neutron PDF paper on $LiNiO_2$ such as the trimer, dimer, and zigzag ordering.

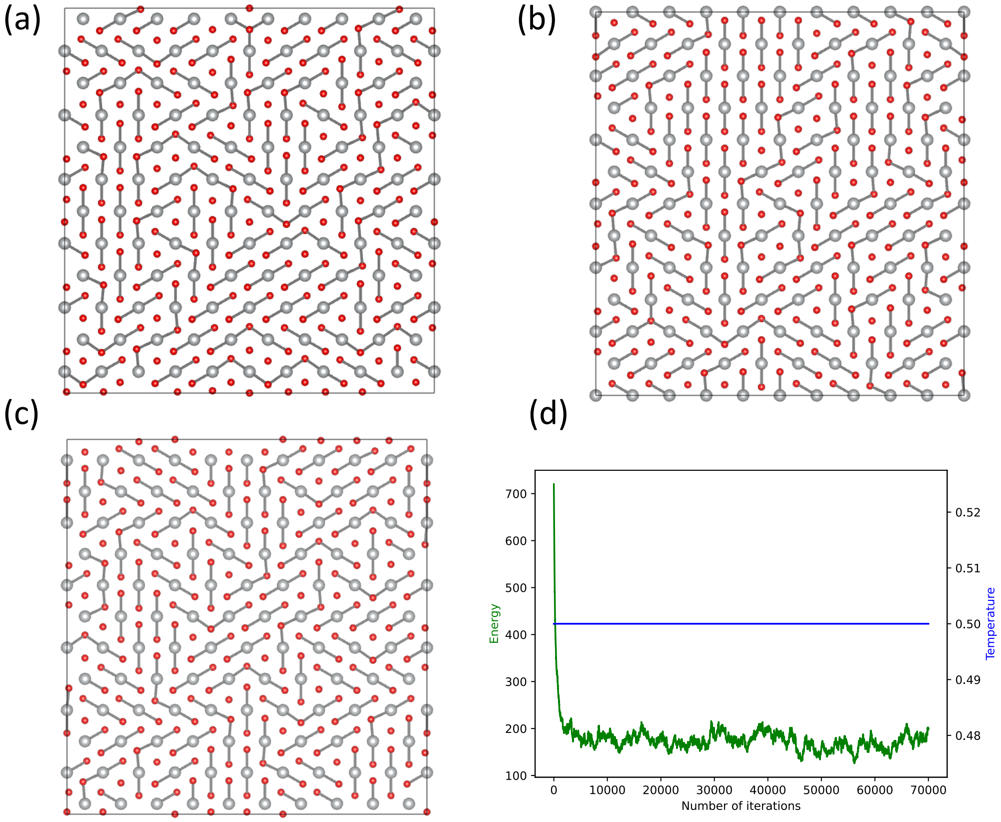

Figure S17: (a,b,c) Example O-Ni-O layers, and (d) energy dependence on cycle, for generating locally JT-distorted $10 \times 6 \times 1$ supercells. In this case, starting $E_{\text{total}} = 718$; final $E_{\text{total}} = 200$ (as defined in Equation S6). Grey atoms are Ni and red atoms are O. Only JT-elongated Ni-O bonds are shown.

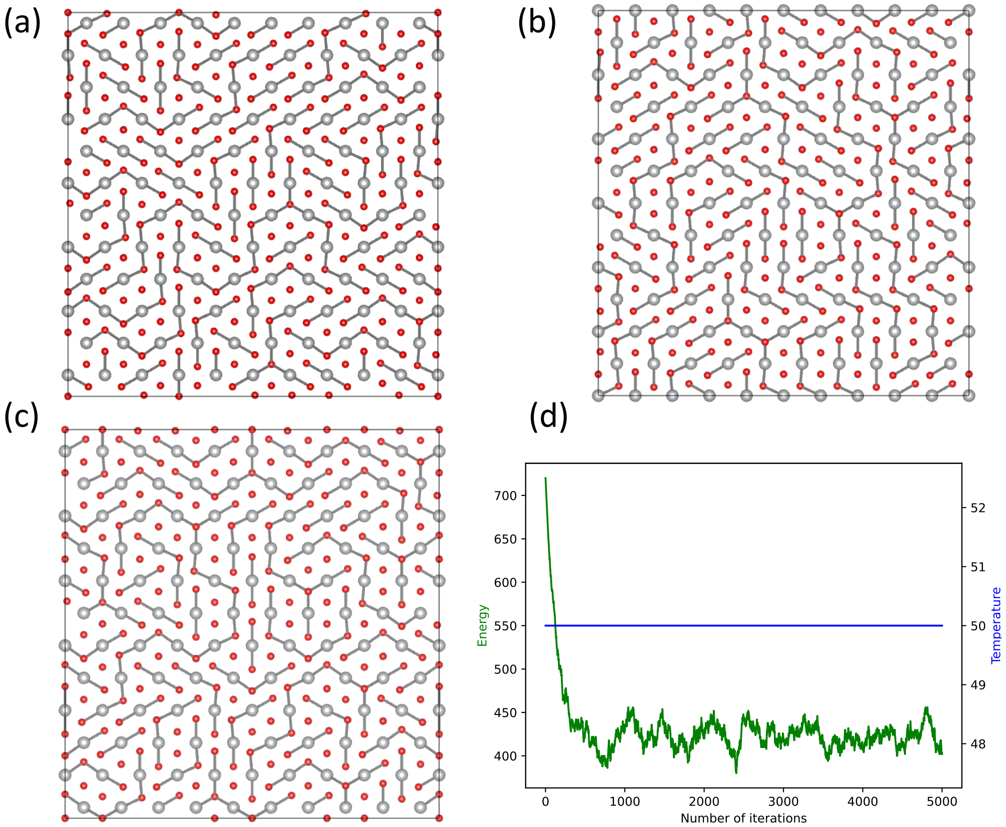

Figure S18: (a,b,c) Example O-Ni-O layers, and (d) energy dependence on cycle, for generating locally JT-distorted $10 \times 6 \times 1$ supercells. In this case, starting $E_{\mathrm{total}} = 718$; final $E_{\mathrm{total}} = 200$ (as defined in Equation S6). Grey atoms are Ni and red atoms are O. Only JT-elongated Ni-O bonds are shown.

S3.7.2 Results from big box fitting from JT-distorted starting configuration

Next, we present the results of big box refinements using a $16 \times 9 \times 3$ supercell. For these we use the same restraints as in the other results presented in this work, and we repeat each run with no BVS restraints. We have performed several refinements using different temperatures and different numbers of iterations during the Monte Carlo run. For each big box refinement, we used VanVleckCalculator[2] to obtain the bond length distortion index and $\rho_0 = \sqrt{Q_2^2 + Q_3^2}$. This is shown in Table S2. Associated bond length distributions are shown in Figures S19 and S20. Probability distributions of ρ_0 are shown in Figure S21.

The fits without BVS restraints show a disappearance of local JT distortions, converging on a similar structure to that obtained from a starting configuration without local JT distortions, as shown elsewhere in this work.

However, we find that fits performed with BVS restraints actually retain some local elongations, although this is very subtle. It cannot clearly be discerned from the bond length histograms [Figure S19] and is only clear when looking at the probability distribution of ρ_0 [Figure S21(a)], where the maximum probability typically occurs when ρ_0 is slightly larger than 0 Å, around 0.15 Å.

This means we cannot rule out the possibility for very small, local JT distortions in $NaNiO_2$, but we note that the BVS penalties actually drive local JT distortions in a disordered JT-distorted starting structure [Figure S21(b)], and so this may just be the result of the BVS restraints.

Table S2: Outputs of big box refinements against neutron pair distribution function data, following the same approach as described in the main text, but varying the starting configuration using the Monte Carlo approach described here. BLDI is bond length distortion index[37] and $\rho_0 = \sqrt{Q_2^2 + Q_3^2}$.[2] BLDI and ρ_0 are given to a level of precision set by the standard deviation for all octahedra. R_{wp} is given to 2 decimal places. For interpreting the R_{wp} values given, note that the R_{wp} for the big box fit presented in the main text (with BVS restraints applied and without Monte Carlo-generated starting configurations) are 4.10 and 3.72 for the real- and reciprocal-space data, respectively.

Monte-Carlo details				Refinement outputs			
T (T_c)	Num. of iterations	final E_{total} (T_c^{-1})	BVS restraints	BLDI	ρ_0 (Å)	R_{wp} (PDF)	R_{wp} (Bragg)
0.5	10,000	1616	On	0.044 ± 0.014	0.19 ± 0.08	4.20	3.69
			Off	0.045 ± 0.017	0.14 ± 0.07	4.94	3.86
0.5	100,000	1234	On	0.045 ± 0.014	0.20 ± 0.08	4.15	3.69
			Off	0.042 ± 0.013	0.16 ± 0.07	5.83	3.82
5	10,000	2858	On	0.045 ± 0.014	0.20 ± 0.08	5.41	3.79
			Off	0.046 ± 0.017	0.15 ± 0.07	5.72	3.82
5	25,000	2922	On	0.045 ± 0.015	0.19 ± 0.09	4.44	3.69
			Off	0.042 ± 0.014	0.15 ± 0.07	5.70	3.80
50	4,500	3098	On	0.045 ± 0.015	0.19 ± 0.09	4.27	3.69
			Off	0.044 ± 0.015	0.15 ± 0.07	5.56	3.87
50	10,000	3070	On	0.045 ± 0.014	0.20 ± 0.08	4.33	3.69
			Off	0.048 ± 0.018	0.16 ± 0.07	5.52	3.80

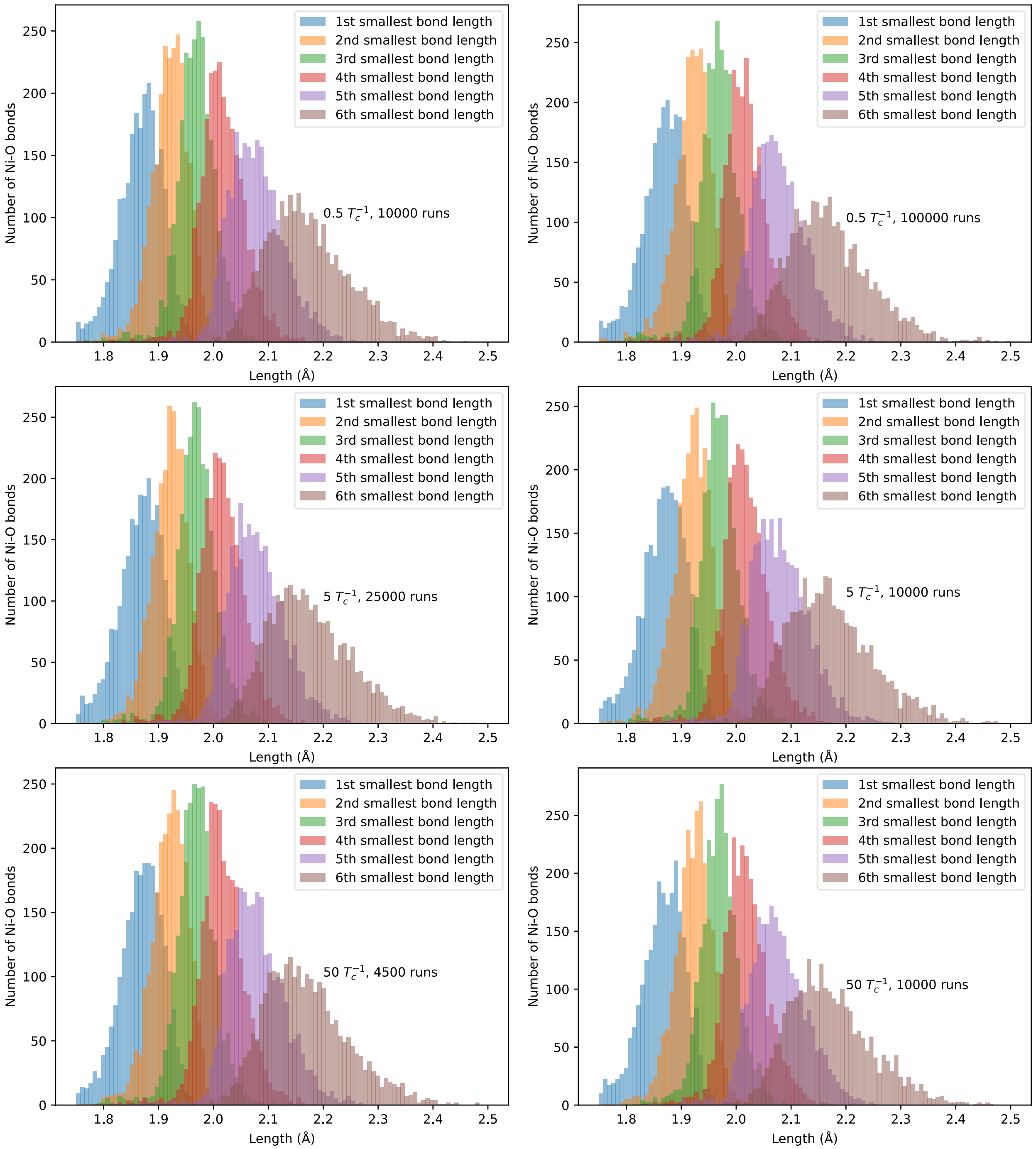

Figure S19: Ni-O bond length distributions for big box analysis of 500 K neutron PDF data, using starting configurations with random Jahn–Teller distortions. Each distribution corresponds to a particular configuration in Table S2 with BVS restraints enabled.

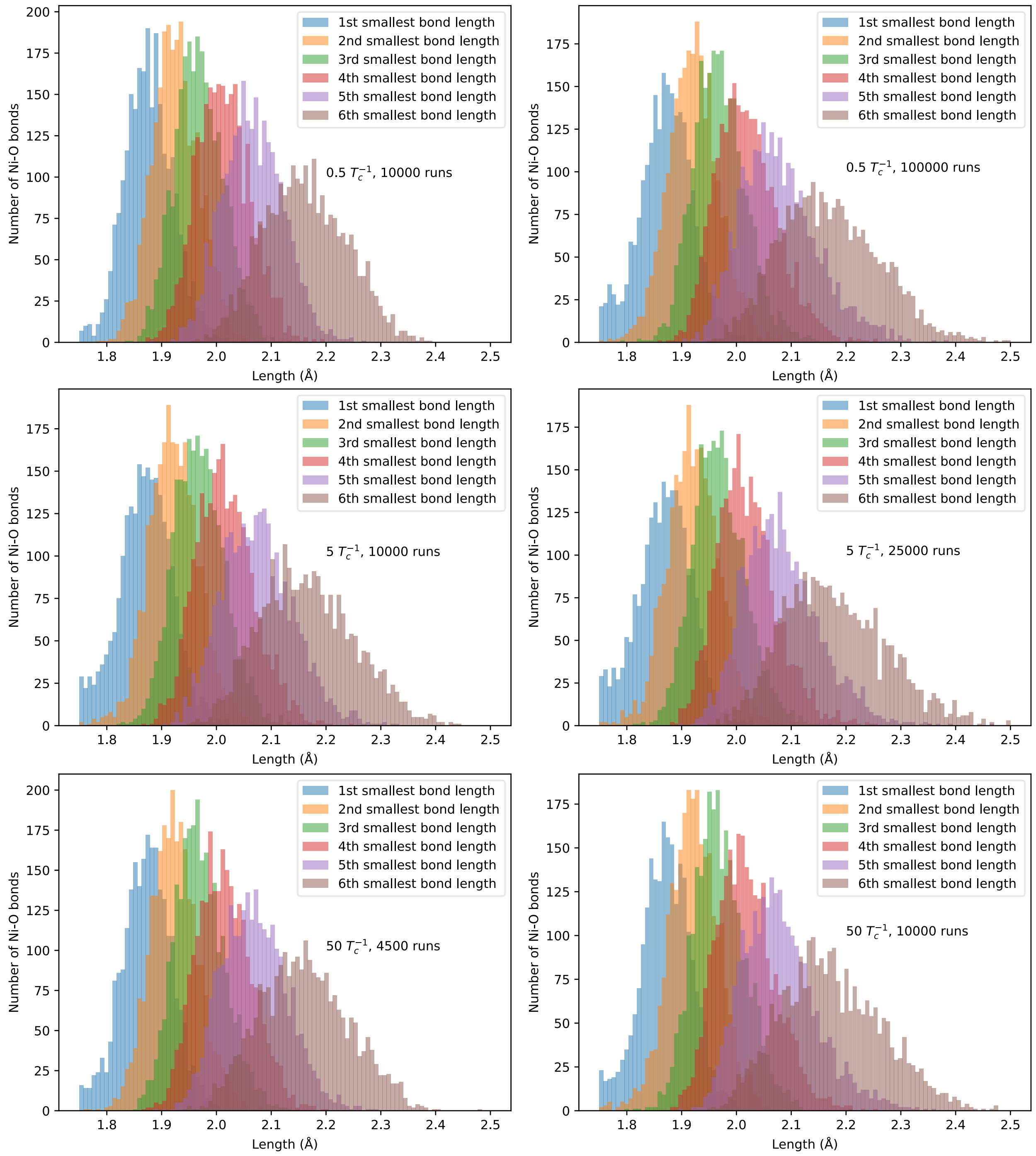

Figure S20: Ni-O bond length distributions for big box analysis of 500 K neutron PDF data, using starting configurations with random Jahn–Teller distortions. Each distribution corresponds to a particular configuration in Table S2 with BVS restraints not enabled. This is the counterpart to Figure S19 which shows the case where BVS restraints are enabled.

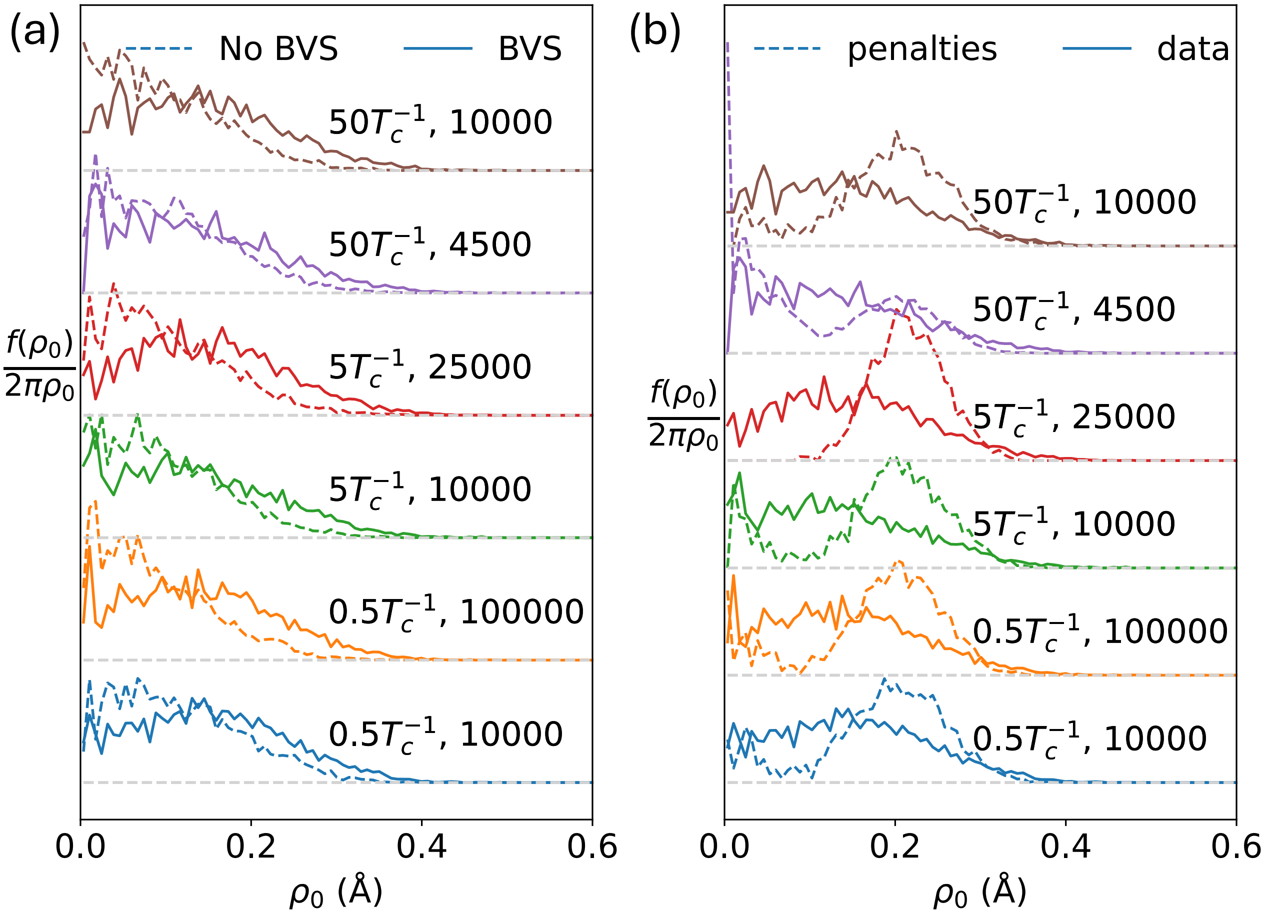

Figure S21: Probability distributions of ρ_0 for the big box PDF analysis from Monte Carlo-generated starting structures, as outlined in Table S2. (a) Distributions for the refinements with and without BVS restraints, refined against the data. (b) Distributions from fitting with the BVS restraints against the data, with a refinement performed solely against the BVS penalties; this shows that here the BVS restraints actually bias in favour of local JT distortions when the starting configuration is JT-distorted.

S3.8 Visualising the Ni-O bond lengths from big box neutron PDF analysis in terms of short and long bonds

The fitting of the JT-distorted (named {2,...} models in the notation in this work, indicating the presence of 2×Ni-O bonds) to the EXAFS data suggests a narrowing of the distance between the short and long Ni-O bonds at high-temperature, once the average structure becomes rhombohedral. This could be interpreted as indicating the Jahn–Teller distortion persists locally, but at a reduced magnitude, within the rhombohedral phase. This is however not our interpretation, for reasons outlined in the manuscript; we believe the JT distortion is displacive and hence there will not be local JT distortions at 500 K. As a further aside, we note that fitting a 4×short and 2×long bond lengths to a peak with an asymmetric bond length distribution (as is most clearly shown in the PDF data at 500 K) will give a short and long bond length exhibiting this behaviour regardless of whether there is a tetragonal elongation of the octahedron.

To show this, we plot in Figure S22 histograms of the bond lengths from the big box PDF analysis in this study, where we have taken the average of the 4 short bonds and the average of the 2 long bonds in each octahedron. In this way, we obtain results which are most clearly comparable to the EXAFS data. Noting that the big box PDF analysis clearly supports a displacive JT transition and the absence of a JT effect at 500 K, we see a very similar trend where there is a long and short Ni-O bond length, and the difference between long and short is reduced at 500 K compared with 293 K and 450 K. If viewed alone, this could be interpreted similarly to the EXAFS, meaning that one could conclude the big box PDF analysis supports local, reduced JT distortions at 500 K. When viewed in combination with other evidence, such as Figure 5(a) in the main paper, this interpretation no longer seems viable.

We include this as evidence that the Ni-O bond lengths obtained by fitting the JT-distorted model to the EXAFS data can be explained in other ways besides a local JT distortion at high-temperature.

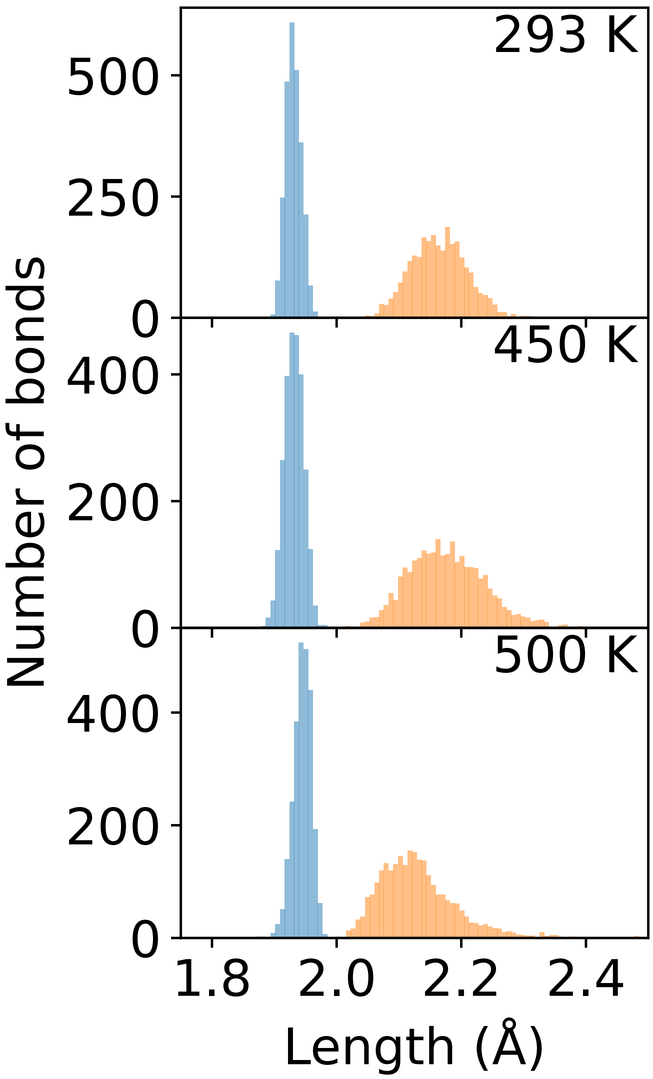

Figure S22: Histogram of Ni-O bond length from big box neutron PDF analysis, presented as the average of the 4 smallest and 2 longest bonds per octahedron. This shows that, despite clear evidence for the absence of Jahn–Teller distortions at 500 K presented elsewhere in this manuscript, averaging different bonds within bond length distributions in terms of a particular splitting will present misleading results in support of that particular bond splitting. This is important for interpreting the EXAFS data as fit with the {2}, {2,1}, and {2,2} models.

S3.9 Big box analysis without BVS restraints

The TOPAS-based[38] big box analysis of pair distribution function data performed in this study utilised bond valence sum (BVS) restraints[39] to ensure reasonable behaviour of coordination octahedra. Precise details on this are described in the methods section of the main paper.

Here, we present alternatively the results of refinements against the pair distribution function data, following an identical method to that described in the main paper except no bond valence sum restrains were applied. Overall, the findings are qualitatively consistent with those presented in the main paper, in the sense that there is no evidence for a persistent elongational Jahn–Teller distortion at 500 K; see Figure S23. The fit is slightly improved in quality compared with the fit obtained with BVS restraints (see Table S3 and Figure S24). With BVS restraints, we obtained a slightly negative Q_3, although the deviation between the mean Q_3 and $Q_3 = 0$ was far smaller than the standard deviation in Q_3, where $\bar{Q_3} = (0.02 \pm 0.12)$ Å; in contrast, the refinements without BVS show a far more negative value of $\bar{Q_3} = (-0.08 \pm 0.09)$ Å, which is a far more significant deviation from $Q_3 = 0$ towards a tetragonal compression. A histogram of Q_3 analogous to Figure 5(c) in the main paper, but without BVS restraints, is shown in Figure S25. To ensure that this shift to negative Q_3 is not caused by the floating origin restraint (as discussed in methods, shifts in the average Ni position are restrained due to the floating origin of the $P1$ space group), we tested the refinement using the ONLY_PENALTIES flag in TOPAS without BVS restraints using the 500 K starting cell and we did not find a negative Q_3, so this effect is not the consequence of penalties. We also show the probability distribution of ρ_0 in Figure S26, which is very similar to the case with BVS applied (as shown in the main paper).

In Figure S23, we see that Ni-O bond lengths exhibit much larger tails into the physically unfeasible low- or high-bond lengths, without BVS restraints. This still occurs with BVS restraints (see Figure 5(a) in the main paper) but to a much lesser extent. This may be due to overfitting of the model to features of the data occurring due to the Fourier transform,

i.e. Fourier ripples. In Figure S9(c,d), we showed the bond length width vs average length for each bond, and the issue with over-fitting of low-r tails can be seen from the magnitude of the standard deviation for each bond length.

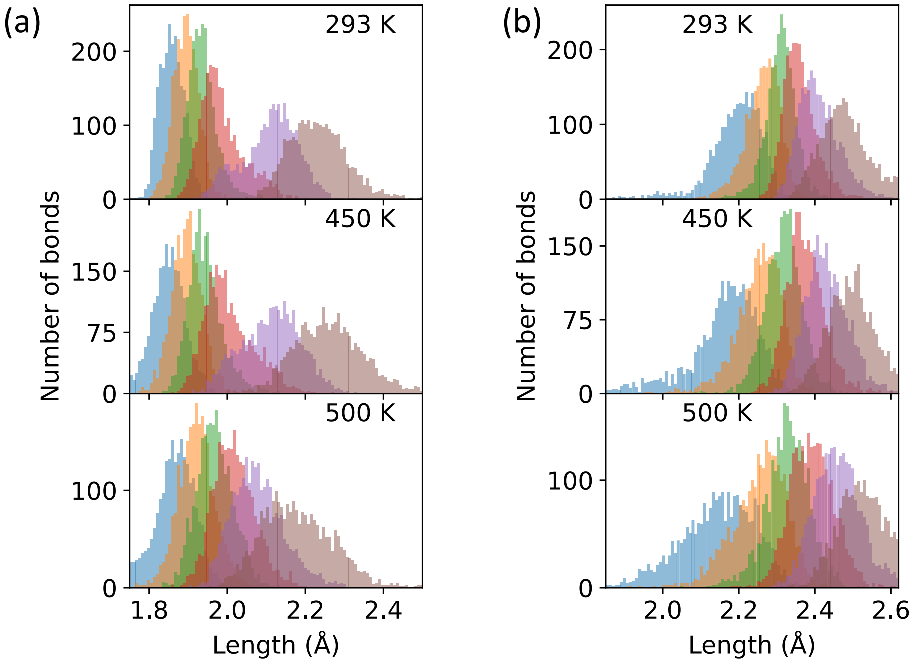

Figure S23: Histogram of (a) Ni-O and (b) Na-O bond lengths from big box neutron PDF analysis, where BVS restraints were not applied. Each distribution at a given temperature refers to the smallest to largest bond length in an octahedron, depending on its relative position in the plot.

Table S3: Comparison of R_{wp} from fitting the big box model to the neutron PDF and Bragg data, with and without bond valence sum restraints. Values are rounded to 2 decimal places.

	with BVS		without BVS	
T	R_{wp} (PDF)	R_{wp} (Bragg)	R_{wp} (PDF)	R_{wp} (Bragg)
293 K	7.45	3.88	4.66	3.39
450 K	6.01	3.45	5.65	3.23
500 K	4.10	3.72	5.41	3.79

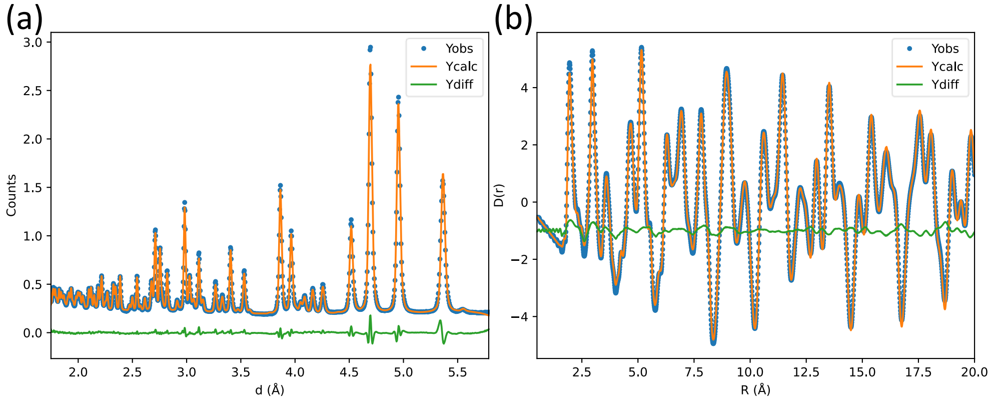

Figure S24: (a) Bragg and (b) PDF neutron diffraction data, fit via a big box method where BVS restraints were not applied, to the data at 500 K.

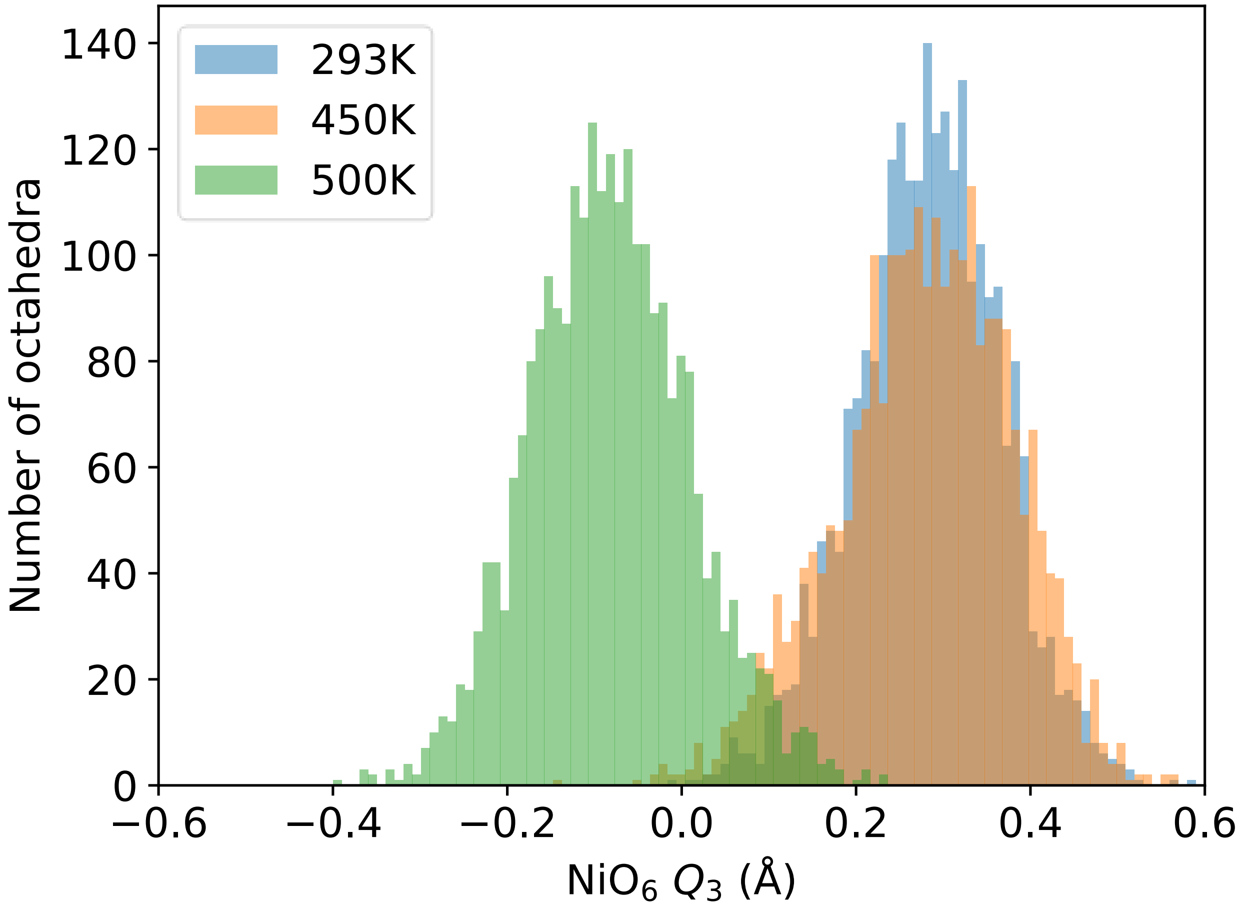

Figure S25: A histogram of the distribution of calculated NiO_6 Q_3 values at 293 K, 450 K, and 500 K, obtained via big box analysis where BVS restraints were not applied. As discussed in the main text, we tested to see if the shift to negative Q_3 at 500 K is due to the floating point restraint and we found that it is not.

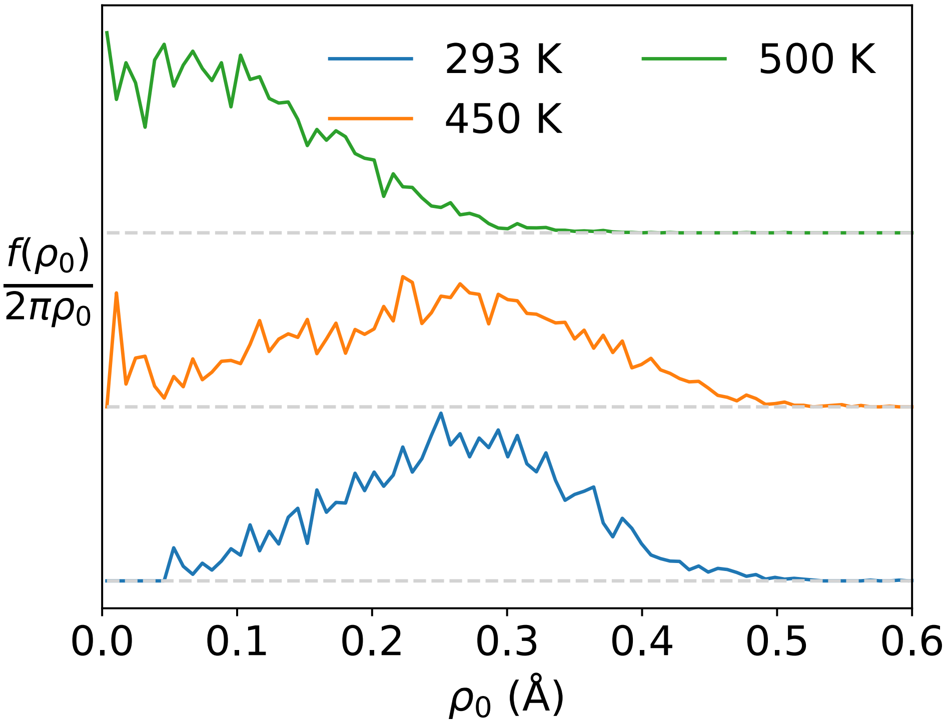

Figure S26: A histogram of the probability distribution of ρ_0 obtained via big box analysis where BVS restraints were not applied.

S3.10 Trans-octahedral O-O distances from big box PDF analysis

In the manuscript, we primarily use the Ni-O distances, and associated parameters such as bond length distortion index and van Vleck modes, to quantify the Jahn–Teller distortion. However, many works alternatively use the trans-octahedral O-O distance (i.e. O-O distances whose Ni-O bond lengths are at a 180° angle to one another). Here, in Figure S27, we present histograms of trans-octahedral O-O bond lengths from the supercells output by big box PDF. While we performed several big box analyses in this study, these histograms relate to the same supercells as those for which histograms of Ni-O bond lengths and bond length distortion index are presented in the main paper.

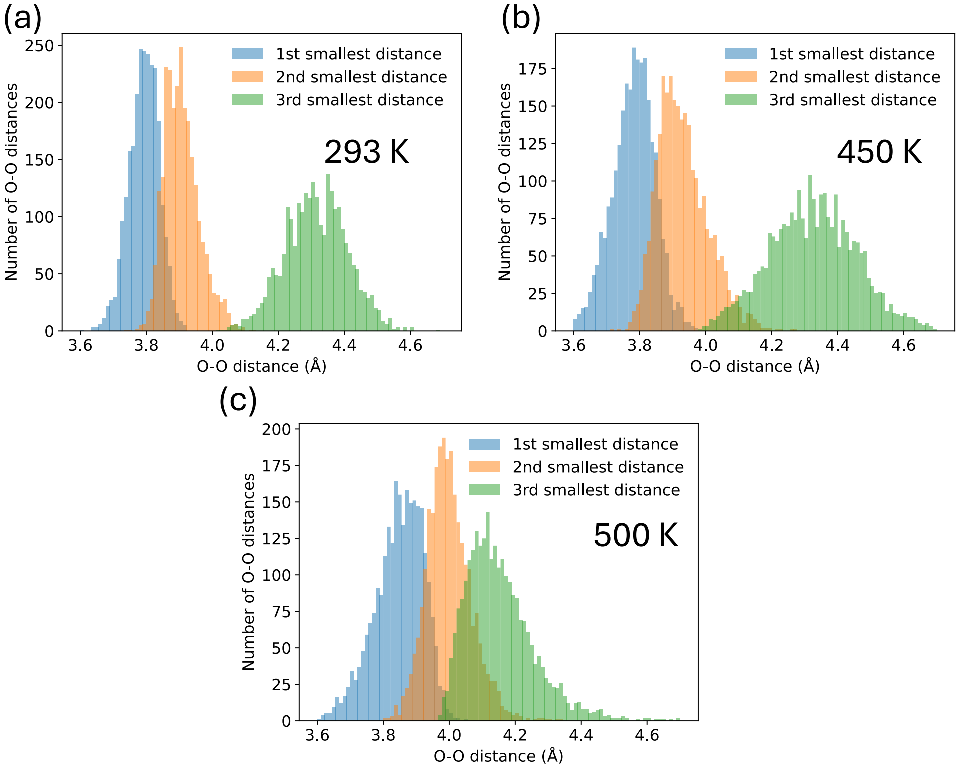

Figure S27: Histogram of trans-octahedral (NiO_6 octahedra) O-O distances from big box neutron PDF analysis, at (a) 293 K, (b) 450 K, and (c) 500 K. Each distribution at a given temperature refers to the smallest to largest O-O distance length in an octahedron, depending on its relative position in the plot. Trans-octahedral means O-O distances that go via the centre of the octahedron. These histograms relate to the same supercells as those for which histograms of Ni-O bond lengths and bond length distortion index are presented in the main paper.

S3.11 Repeating refinements at 500 K for repeatability

In Figure 5(c) of the main paper we show the big box PDF results in terms of the probability distribution $P(\rho_0)$ defined in Equation 1 of the manuscript. We see that the maximum probability occurs at $\rho_0 \approx 0$. To check that this is a repeatable result and not a local minimum, we have repeated the refinement several times, with an identical starting configuration and set of restraints, and found consistency in this result [Figure S28].

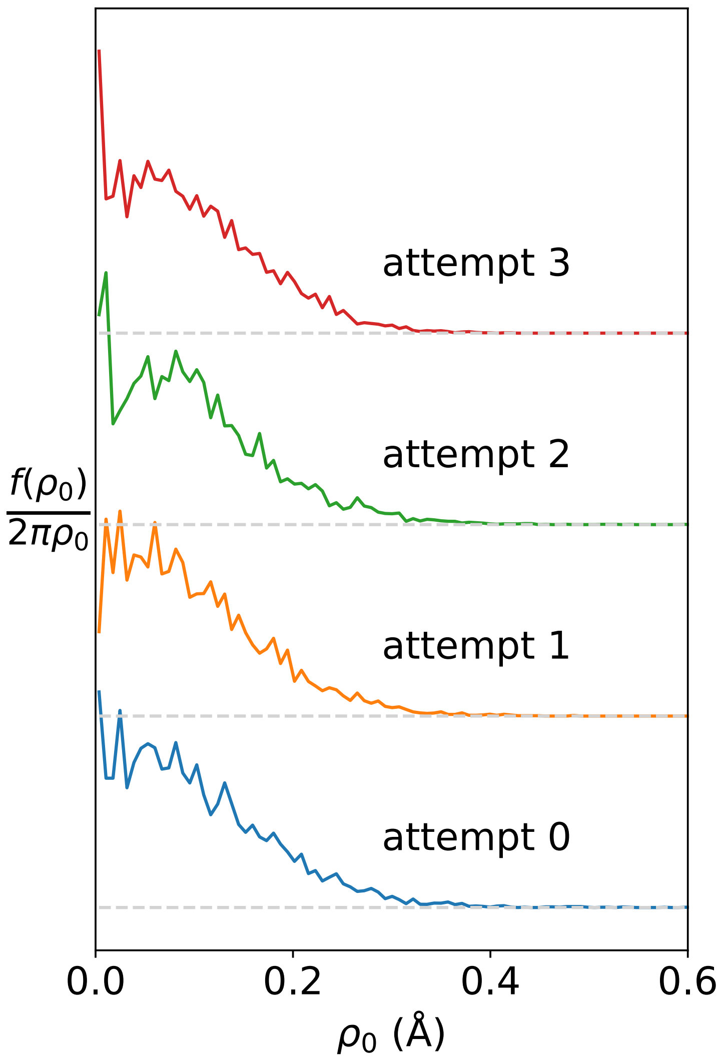

Figure S28: Probability distributions $P(\rho_0)$ for several repeats of the 500 K big box PDF analysis without JT-distorted starting configurations. Note, attempt 0 is the same data as is presented in Figure 5(c) in the main text.

S4 X-ray absorption spectroscopy: additional figures

S4.1 Derivative of μ with energy

Figure S29 shows a plot of $d\mu/dE$ against energy E around the Ni K edge, for the XAS data at several temperatures during heating. The plot shows the pre-peak feature which is more prominent in the monoclinic phase than the rhombohedral phase, but which is barely visible in a plot of $\mu(E)$ against energy E, as shown in the main paper, Figure 6.

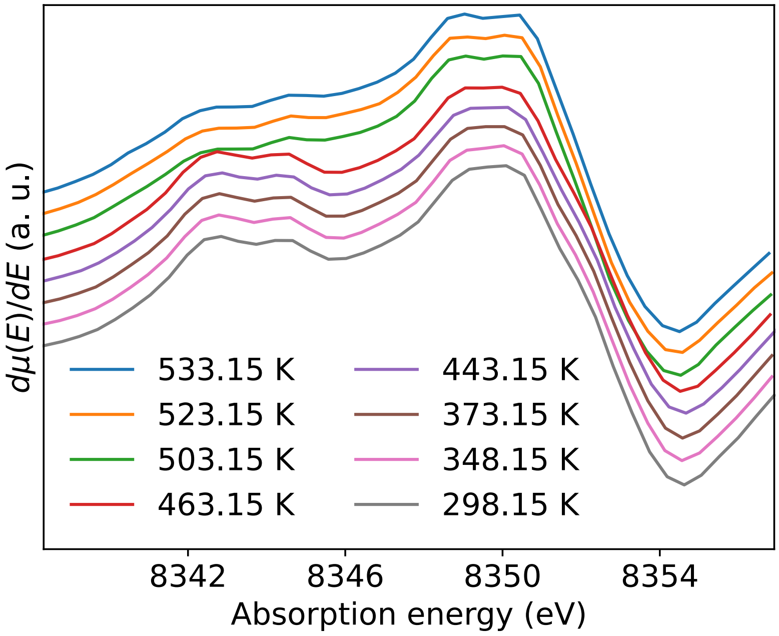

Figure S29: $d\mu/dE$ for the X-ray absorption data around the Ni K edge, with temperature.

S4.2 EXAFS fitting

The EXAFS fitting for the five models had figures of merit shown in Figure S30 during heating. Figure S31 shows the Ni-Ni interatomic distances obtained from fitting the {2,2} model to the EXAFS data during heating.

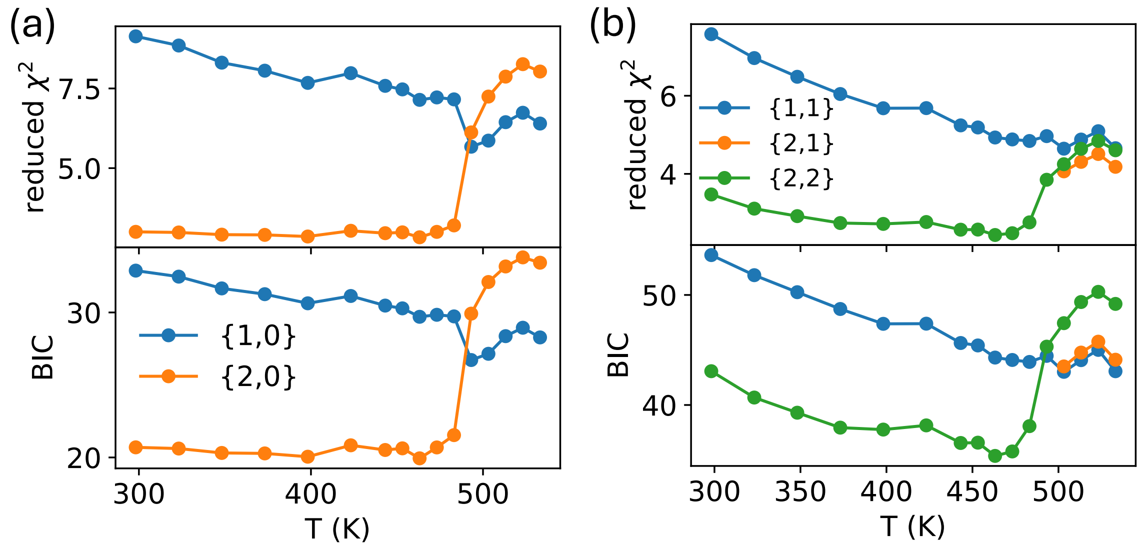

Figure S30: Temperature-dependence of the figures of merit for the 5 different models fit to the EXAFS data. (a) Models {2,0} and {1,0} were fit in the range 0.5 Å to 2.0 Å and (b) {1,1}, {2,1}, and {2,2} were fit in the range 0.5 Å to 3.1 Å. BIC refers to Bayesian Information Criterion.[40]

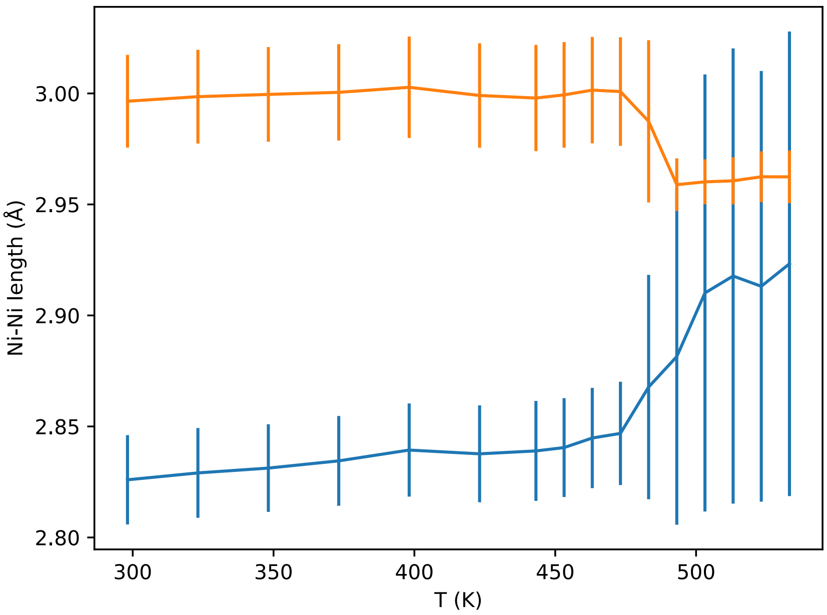

Figure S31: Temperature-dependence of the Ni-Ni interatomic distances obtained by fitting the JT-distorted {2,2} model to the EXAFS data.

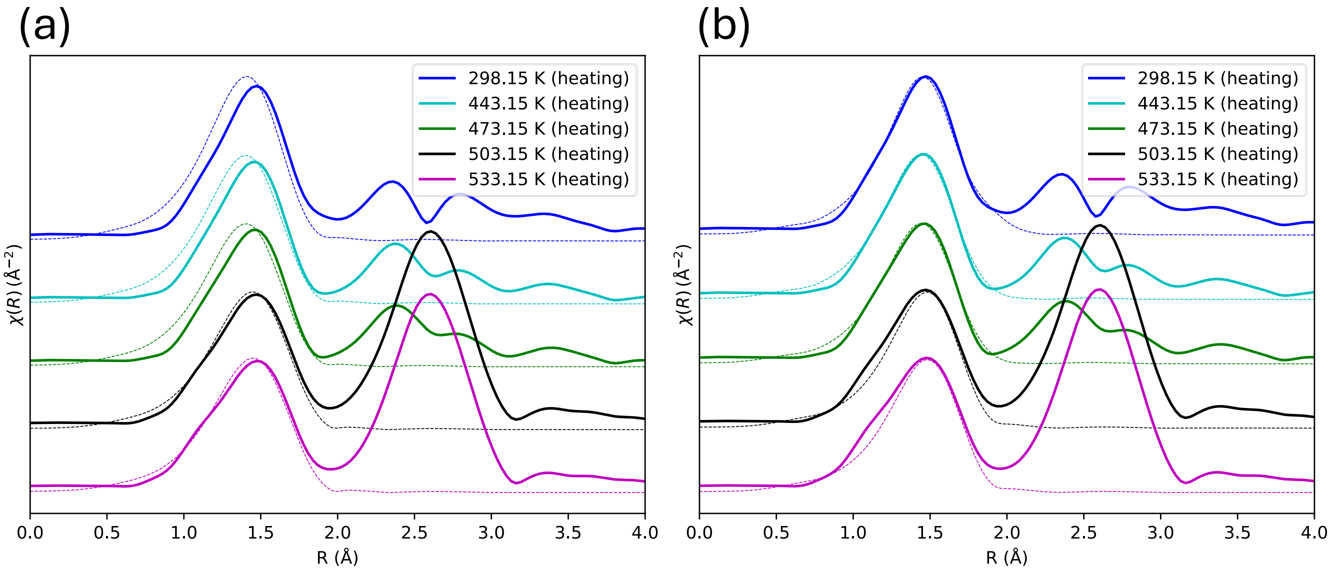

Figure S32: Example EXAFS fits using (a) JT-undistorted {1,0} and (b) JT-distorted {2,0} models at the Ni-O peak. Fits were performed in the range 0.5 Å to 2.0 Å. Solid line is experimental data, dashed line is calculated data.

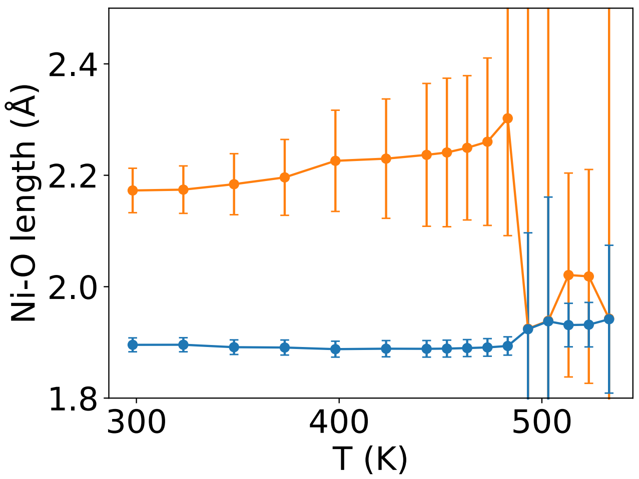

Figure S33: Ni-O bond lengths from EXAFS data, using JT-distorted {2,0} model.

S5 *Ab initio* molecular dynamics: additional plots

S5.1 $E_g(Q_2, Q_3)$ van Vleck plots

Figures S34 and S35 show the Van Vleck $E_g(Q_2, Q_3)$ plots for the *ab initio* molecular dynamics simulations during heating and cooling. These 2D histograms show the calculated modes for all octahedra at all timesteps throughout the simulation, using a $C2/m$ starting cell with collinear JT ordering and an $R\bar{3}m$ JT-undistorted starting cell, respectively.

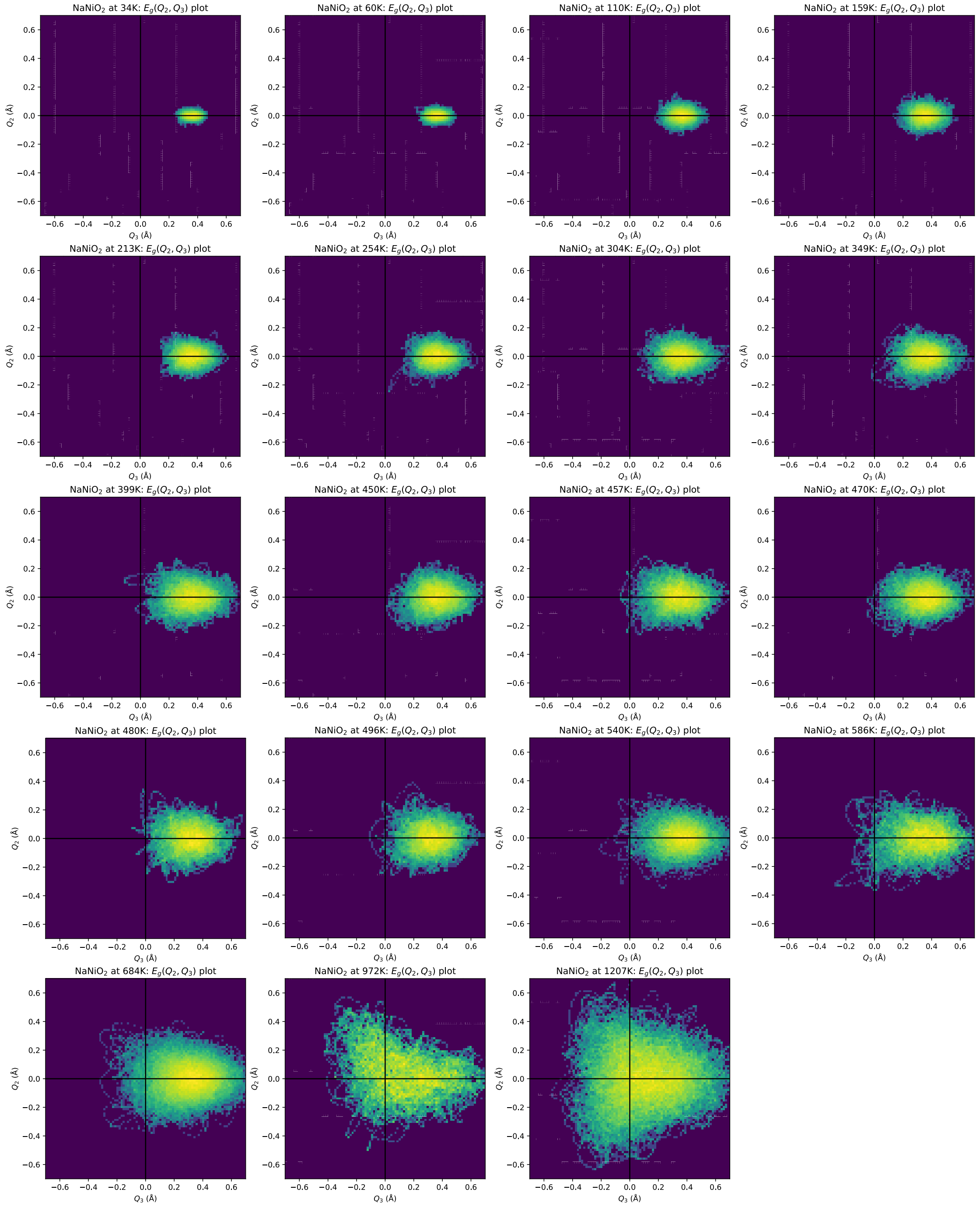

Figure S34: Van Vleck $E_g(Q_2, Q_3)$ plots for the *ab initio* molecular dynamics simulations during heating. These 2D histograms show the calculated modes for all octahedra at all timesteps throughout the simulation, using a $C2/m$ starting cell with collinear JT ordering.

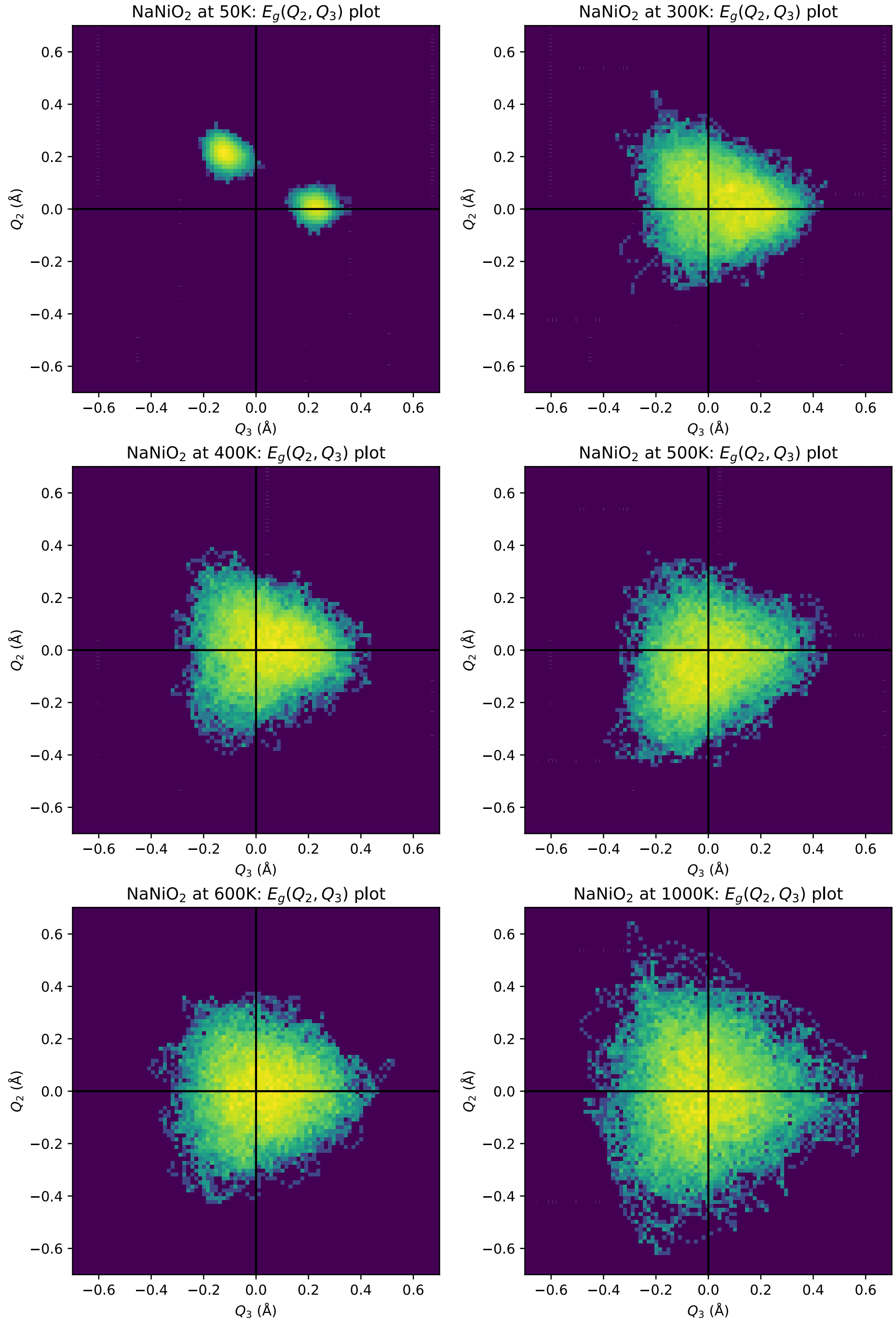

Figure S35: Van Vleck $E_g(Q_2, Q_3)$ plots for the *ab initio* molecular dynamics simulations during cooling. These 2D histograms show the calculated modes for all octahedra at all timesteps throughout the simulation, using a $R\bar{3}m$ starting cell with no JT distortion. In (a), we obtain a row-ordered model as in Ref.[41] which is not expected; this is probably a result of rapid quenching as described in the main text.

S5.2 Convoluting histograms of bond lengths with $Q_{\max}$

In the main paper, Figure 8, we presented a histogram of Ni-O bond lengths at several temperatures from the AIMD simulation cells. In this section, we convolute this histogram with a sinc function using the experimental $Q_{\max} = 40\,\text{Å}^{-1}$ to demonstrate that the differences shown by AIMD can be resolved by the experiment we performed.

This convolution was based on the following function derived in Appendix B of Chung and Thorpe (1997):[42]

$$G_e(r) = \frac{1}{\pi}\int_0^{\infty} G'(r)\left[\frac{\sin\left[Q_{\max}(r-r')\right]}{r-r'} - \frac{\sin\left[Q_{\max}(r+r')\right]}{r+r'}\right] dr' \qquad \text{(S8)}$$

where $G_e(r)$ is the convolved histogram of bond lengths and $G'(r)$ is the raw histogram calculated from the AIMD simulation cells. In practice, a summation was used rather than an integral due to the finite r range over which a histogram was obtained, and the discrete r spacings.

Figure S36 shows the results of this convolution at some representative temperatures. It shows that the predictions of AIMD can be distinguished via the neutron PDF experiment performed in this work.

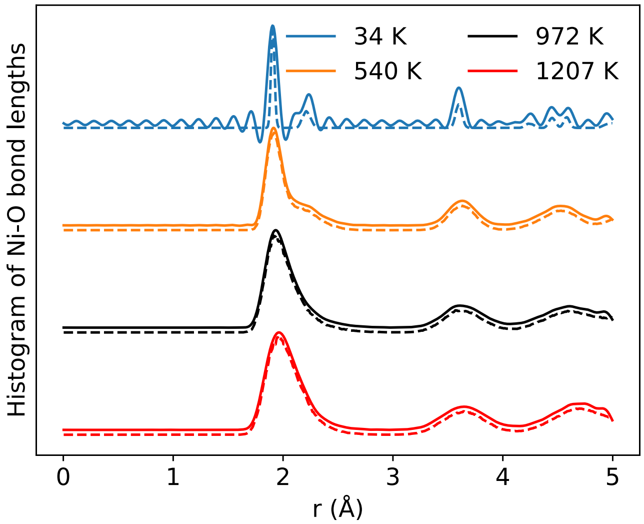

Figure S36: Histograms of Ni-O bond lengths from AIMD convolved with experimental Q_{max} using Equation S8. Solid lines show the convolved dataset and dashed line is the raw Ni-O histogram from the AIMD structures. A small vertical offset is artificially applied between the convolved dataset and raw dataset at each temperature, so these can more easily be distinguished. Note that no scattering lengths are used and so peak intensities are not scaled as they would be in neutron/X-ray scattering. The height of each dataset is normalised to be equal.

S6 ^{23} Nuclear Magnetic Resonance figure

In the main paper, variable-temperature nuclear magnetic resonance was discussed. We present the data here, in Figure S37.

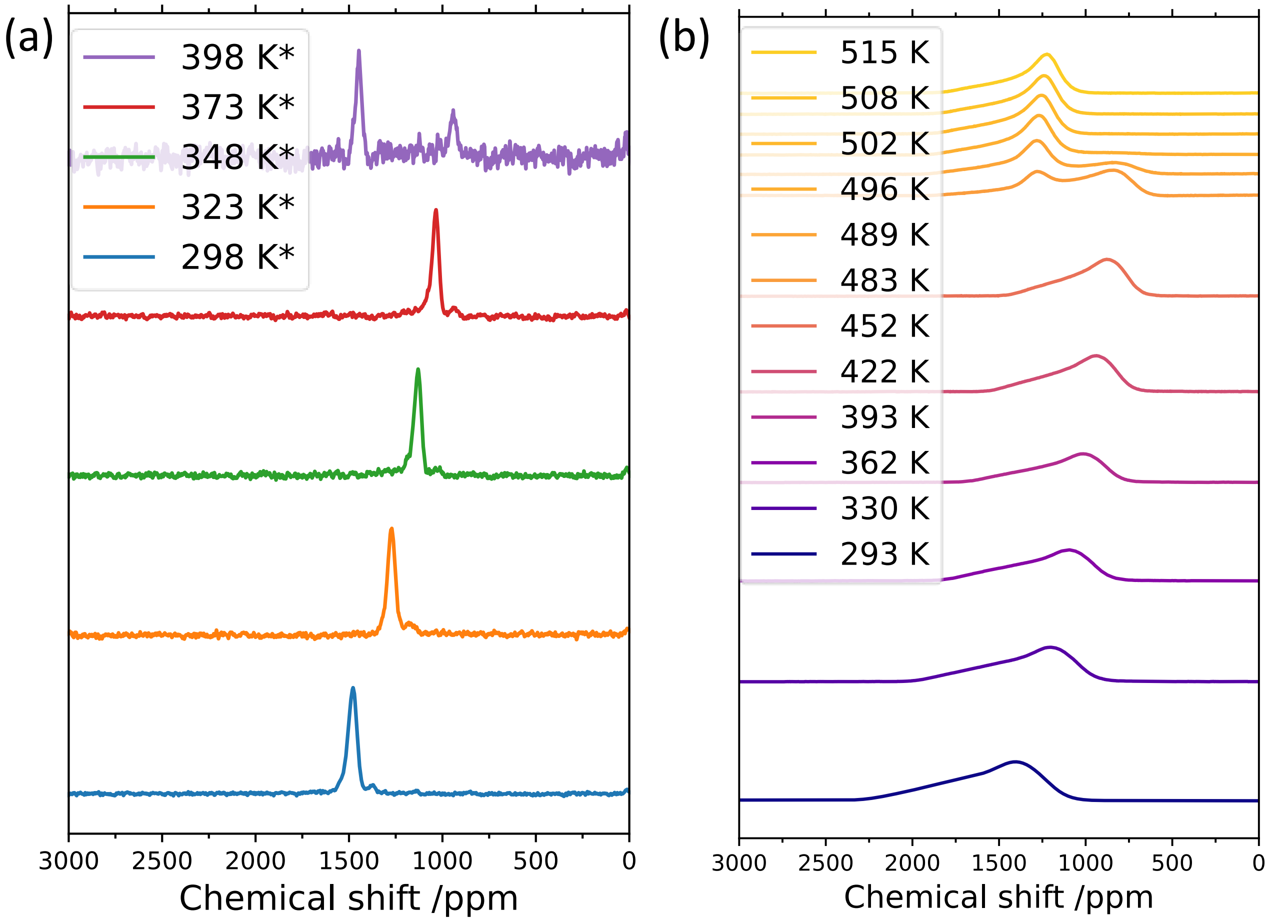

Figure S37: (a) ^{23}Na ssNMR pj-MATPASS (14 kHz) spectra of $NaNiO_2$ with increasing temperature. Note that temperatures (*) were not calibrated, and thus are not reflective of the true sample temperature. (b) ^{23}Na ssNMR Hahn-echo (static) spectra of $NaNiO_2$ with increasing temperature. The initial monoclinic environment (dark purple line), and emergent rhombohedral environment (orange line) are displayed.

S7 Tabulated data

S7.1 Big box neutron PDF: lattice parameters of supercell

Table S4 shows the lattice parameters with temperature based on refinements against the supercell.

	Unit cell						Supercell					
T (K)	a (Å)	b (Å)	c (Å)	α (°)	β (°)	γ (°)	a (Å)	b (Å)	c (Å)	α (°)	β (°)	γ (°)
293	2.846378	5.320907	15.70395	88.05271	90	90	45.54205	47.88816	47.11186	88.05271	90	90
450	2.853097	5.330341	15.75371	88.06143	90	90	45.64955	47.97307	47.26113	88.06143	90	90
500	2.964317	5.134347	15.77657	90	90	90	47.42907	46.20912	47.32971	90	90	90

Table S4: The lattice parameters of the pseudo-orthorhombic unit cell and $16 \times 10 \times 3$ supercell refined against the neutron Pair Distribution Function data in this study.

S7.2 Big box neutron PDF: octahedral properties for supercell refined against experimental data

In this subsection, the average and standard deviation of various properties of the octahedra in the supercell are tabulated, as obtained by big box refinements against the Bragg and PDF data (for details, see the main paper). Table S5 shows this for the NiO_6 octahedra and Table S6 shows the equivalent for the NaO_6 octahedra.

T (K)	Q_2 (Å)		Q_3 (Å)		ρ_0 (Å)		BLDI		Volume (Å^3)		BAV ($^{\circ 2}$)		BL (Å)	
	$\bar{X}$	σ	$\bar{X}$	σ	$\bar{X}$	σ	$\bar{X}$	σ	$\bar{X}$	σ	$\bar{X}$	σ	$\bar{X}$	σ
293	-0.0044	0.0702	0.2673	0.0670	0.2770	0.0647	0.0547	0.0126	10.291	0.117	58.4	38.6	2.009	0.127
450	-0.0013	0.0956	0.270	0.102	0.2892	0.0949	0.0575	0.0162	10.284	0.142	61.9	28.9	2.013	0.138
500	0.0007	0.1310	-0.030	0.126	0.1618	0.0877	0.0458	0.0182	10.291	0.180	70.8	32.5	2.007	0.120

Table S5: NiO_6 octahedral properties in the supercell obtained by big box refinement against neutron PDF data. Properties were calculated using VanVleckCalculator.[2] Q_2 and Q_3 are the van Vleck modes,[1,2] and $\rho_0 = \sqrt{Q_2^2 + Q_3^2}$. BLDI refers to the bond length distortion index,[37] volume refers to the octahedral volume which is calculated by decomposing the octahedra into 8 tetrahedra, BAV refers to the bond angle variance,[37] and BL refers to the Ni-O bond lengths. $\bar{X}$ and σ refer to the mean and standard deviation for all octahedra respectively. All values are listed to the third significant figure of σ.

T (K)	Q_2 (Å)		Q_3 (Å)		ρ_0 (Å)		BLDI		Volume (Å^3)		BAV ($^{\circ 2}$)		BL (Å)	
	$\bar{X}$	σ	$\bar{X}$	σ	$\bar{X}$	σ	$\bar{X}$	σ	$\bar{X}$	σ	$\bar{X}$	σ	$\bar{X}$	σ
293	0.0030	0.0688	0.0577	0.0768	0.1036	0.0569	0.0260	0.0151	15.958	0.196	174.3	62.3	2.3392	0.0817
450	0.0009	0.0946	0.054	0.103	0.1314	0.0719	0.0352	0.0184	16.068	0.275	185.7	72.8	2.350	0.113
500	-0.002	0.125	0.024	0.124	0.1550	0.0866	0.0450	0.0215	16.128	0.475	188.1	65.0	2.360	0.146

Table S6: NaO_6 octahedral properties in the supercell obtained by big box refinement against neutron PDF data. Properties were calculated using VanVleckCalculator.[2] Q_2 and Q_3 are the van Vleck modes,[1,2] and $\rho_0 = \sqrt{Q_2^2 + Q_3^2}$. BLDI refers to the bond length distortion index,[37] volume refers to the octahedral volume which is calculated by decomposing the octahedra into 8 tetrahedra, BAV refers to the bond angle variance,[37] and BL refers to the Na-O bond lengths. $\bar{X}$ and σ refer to the mean and standard deviation for all octahedra respectively. All values are listed to the third significant figure of σ.

S7.3 Big box neutron PDF: octahedral properties for supercell refined against penalties only

In the big box analysis, penalties are applied to atomic positions deviating from those required to achieve the expected bond valence sum (a proxy for oxidation state) of ions, via the bond valence sum method (for details see the main paper). To test these penalties are behaving reasonably and not overly constraining the data, refinements of the supercell were performed against the penalties only, without including the experimental data, using the TOPAS flag ONLY_PENALTIES. By analogy with the previous section, the properties of octahedra are tabulated here. Table S7 shows this for the NiO_6 octahedra and Table S8 shows the equivalent for the NaO_6 octahedra.

The results shown here differ sufficiently from those in the model achieved by refinement against the data and the penalties, shown in Table S5 and Table S6, that we can conclude that the refinement is not overly constrained.

We should note that lattice parameters were refined in the big box analysis of PDF data, but that refinements in TOPAS using ONLY_PENALTIES do not refine lattice parameters; consequently there are slight variations in lattice parameters between refinements with penalties only compared with data and penalties. Such variations are typically less than 0.01 Å in the subcell (i.e. a single unit of the larger $16 \times 9 \times 3$ supercell) and so these have no significant impact on the findings in this section.

T (K)	Q_2 (Å)		Q_3 (Å)		ρ_0 (Å)		BLDI		Volume (Å^3)		BAV ($^{\circ 2}$)		BL (Å)	
	$\bar{X}$	σ	$\bar{X}$	σ	$\bar{X}$	σ	$\bar{X}$	σ	$\bar{X}$	σ	$\bar{X}$	σ	$\bar{X}$	σ
293	0.087	0.154	-0.052	0.149	0.209	0.111	0.0685	0.0220	10.141	0.374	178.6	87.9	2.029	0.176
450	0.1234	0.0323	-0.0710	0.0326	0.1461	0.0321	0.03085	0.00601	10.3332	0.0337	44.31	5.59	1.9972	0.0740
500	0.0017	0.0346	-0.0002	0.0342	0.0435	0.0218	0.03266	0.00521	10.4085	0.0365	43.37	6.16	1.9983	0.0784

Table S7: NiO_6 octahedral properties in the supercell obtained by big box refinement against the BVS penalties. Properties were calculated using VanVleckCalculator.[2] Q_2 and Q_3 are the van Vleck modes,[1,2] and $\rho_0 = \sqrt{Q_2^2 + Q_3^2}$. BLDI refers to the bond length distortion index,[37] volume refers to the octahedral volume which is calculated by decomposing the octahedra into 8 tetrahedra, BAV refers to the bond angle variance,[37] and BL refers to the Ni-O bond lengths. $\bar{X}$ and σ refer to the mean and standard deviation for all octahedra respectively. All values are listed to the third significant figure of σ.

T (K)	Q_2 (Å)		Q_3 (Å)		ρ_0 (Å)		BLDI		Volume (Å^3)		BAV ($^{\circ 2}$)		BL (Å)	
	$\bar{X}$	σ	$\bar{X}$	σ	$\bar{X}$	σ	$\bar{X}$	σ	$\bar{X}$	σ	$\bar{X}$	σ	$\bar{X}$	σ
293	0.153	0.155	-0.087	0.144	0.247	0.122	0.0626	0.0201	16.001	0.579	263.4	90.6	2.380	0.191
450	0.1372	0.0322	-0.0794	0.0313	0.1617	0.0315	0.03022	0.00649	16.1476	0.0841	150.07	9.81	2.3445	0.0851
500	-0.0013	0.0349	0.0002	0.0341	0.0437	0.0218	0.03239	0.00481	16.2173	0.0755	152.9	11.4	2.3464	0.0919

Table S8: NaO_6 octahedral properties in the supercell obtained by big box refinement against the BVS penalties. Properties were calculated using VanVleckCalculator.[2] Q_2 and Q_3 are the van Vleck modes,[1,2] and $\rho_0 = \sqrt{Q_2^2 + Q_3^2}$. BLDI refers to the bond length distortion index,[37] volume refers to the octahedral volume which is calculated by decomposing the octahedra into 8 tetrahedra, BAV refers to the bond angle variance,[37] and BL refers to the Na-O bond lengths. $\bar{X}$ and σ refer to the mean and standard deviation for all octahedra respectively. All values are listed to the third significant figure of σ.

S7.4 EXAFS data: Figures of merit

To support the conclusions of the main text, figures of merit for all models and sets of paths are shown in Table S9 for the fits to the Ni-O shell only, and in Table S10 for the Ni-O and Ni-Ni shells.

Heating direction	T (K)	χ^2		$r\chi^2$		R-factor		BIC	
		1 × Ni-O	2 × Ni-O	1 × Ni-O	2 × Ni-O	1 × Ni-O	2 × Ni-O	1 × Ni-O	2 × Ni-O
Heating	298.15	90.777	23.82	9.136	3.001	0.167	0.044	32.885	20.698
	323.15	87.91	23.666	8.847	2.982	0.164	0.044	32.47	20.614
	348.15	82.568	23.119	8.309	2.913	0.159	0.045	31.659	20.311
	373.15	80.067	23.061	8.058	2.906	0.155	0.045	31.261	20.279
	398.15	76.262	22.658	7.675	2.855	0.15	0.045	30.631	20.051
	423.15	79.295	24.08	7.98	3.034	0.15	0.046	31.136	20.838
	443.15	75.336	23.485	7.582	2.959	0.149	0.046	30.473	20.515
	453.15	74.236	23.692	7.471	2.985	0.148	0.047	30.283	20.628
	463.15	70.968	22.465	7.142	2.831	0.146	0.046	29.701	19.94
	473.15	71.73	23.809	7.219	3.0	0.145	0.048	29.839	20.692
	483.15	71.123	25.423	7.158	3.203	0.134	0.048	29.729	21.54
	493.15	56.361	48.582	5.672	6.121	0.086	0.075	26.719	29.918
	503.15	58.261	57.505	5.863	7.245	0.082	0.081	27.148	32.099
	513.15	64.011	62.475	6.442	7.872	0.085	0.083	28.366	33.172
	523.15	66.949	65.577	6.738	8.263	0.088	0.086	28.946	33.799
	533.15	63.582	63.741	6.399	8.031	0.087	0.087	28.279	33.431
Cooling	513.15	71.214	70.252	7.167	8.852	0.09	0.089	29.745	34.689
	493.15	73.277	72.615	7.374	9.149	0.089	0.088	30.115	35.117
	473.15	61.5	59.91	6.189	7.549	0.085	0.083	27.848	32.629
	463.15	58.504	33.389	5.888	4.207	0.1	0.057	27.202	25.067
	453.15	67.593	27.155	6.802	3.421	0.12	0.048	29.07	22.393
	443.15	62.173	22.659	6.257	2.855	0.131	0.048	27.989	20.051
	433.15	62.556	21.224	6.295	2.674	0.135	0.046	28.068	19.205
	423.15	67.108	23.298	6.754	2.935	0.139	0.048	28.977	20.411
	413.15	74.63	25.328	7.511	3.191	0.148	0.05	30.351	21.492
	403.15	73.749	23.637	7.422	2.978	0.147	0.047	30.198	20.598
	393.15	73.564	25.134	7.403	3.167	0.151	0.051	30.165	21.392
	373.15	85.738	30.437	8.629	3.835	0.158	0.056	32.146	23.869
	348.15	80.276	26.666	8.079	3.36	0.15	0.05	31.295	22.158
	323.15	96.918	32.075	9.754	4.041	0.161	0.053	33.732	24.547
	298.15	98.55	29.451	9.918	3.711	0.167	0.05	33.948	23.443

Table S9: Figures of merit obtained by fitting a JT-distorted {2,0} (2 × Ni-O) and JT-undistorted {1,0} (1 × Ni-O) model to the EXAFS data as a function of temperature, in the Ni-O shell only (from 0.5 Å to 2 Å). $r\chi^2$ refers to reduced χ^2 and BIC refers to the Bayesian Information Criterion.[40]

Heating direction	T (K)	χ^2			$r\chi^2$			R-factor			BIC		
		{1,1}	{2,1}	{2,2}	{1,1}	{2,1}	{2,2}	{1,1}	{2,1}	{2,2}	{1,1}	{2,1}	{2,2}
Heating	298.15	126.447		44.076	7.576		3.473	0.204		0.071	53.623		43.071
	323.15	116.239		39.471	6.965		3.11	0.192		0.065	51.798		40.678
	348.15	108.244		37.039	6.485		2.919	0.184		0.063	50.252		39.298
	373.15	100.881		34.795	6.044		2.742	0.173		0.06	48.724		37.943
	398.15	94.804		34.511	5.68		2.72	0.164		0.06	47.376		37.765
	423.15	94.864		35.125	5.684		2.768	0.158		0.059	47.39		38.148
	443.15	87.488		32.628	5.242		2.571	0.151		0.056	45.634		36.548
	453.15	86.594		32.67	5.188		2.574	0.15		0.057	45.412		36.576
	463.15	82.306		30.907	4.931		2.436	0.146		0.055	44.31		35.373
	473.15	81.48		31.49	4.882		2.481	0.141		0.055	44.091		35.778
	483.15	80.814		35.015	4.842		2.759	0.122		0.053	43.913		38.079
	493.15	82.893		48.85	4.967		3.849	0.05		0.03	44.464		45.302
	503.15	77.529	59.715	53.903	4.645	4.065	4.248	0.037	0.029	0.026	43.013	43.504	47.437
	513.15	81.424	63.282	58.897	4.879	4.308	4.641	0.036	0.028	0.026	44.076	44.763	49.359
	523.15	84.997	66.242	61.457	5.093	4.509	4.843	0.037	0.029	0.027	45.008	45.754	50.282
	533.15	77.73	61.415	58.432	4.657	4.181	4.605	0.035	0.028	0.026	43.069	44.113	49.187
Cooling	513.15	86.507		65.675	5.183		5.175	0.035		0.027	45.39		51.721
	493.15	92.222		68.669	5.526		5.411	0.035		0.026	46.777		52.688
	473.15	81.858		56.73	4.905		4.47	0.036		0.025	44.192		48.546
	463.15	84.022		44.601	5.034		3.515	0.076		0.04	44.758		43.328
	453.15	81.142		39.616	4.862		3.122	0.107		0.052	44.001		40.757
	443.15	71.814		31.907	4.303		2.514	0.12		0.053	41.352		36.063
	433.15	74.956		31.988	4.491		2.521	0.131		0.056	42.281		36.119
	423.15	78.543		33.2	4.706		2.616	0.135		0.057	43.295		36.925
	413.15	88.643		34.554	5.311		2.723	0.148		0.058	45.919		37.792
	403.15	88.169		33.387	5.283		2.631	0.149		0.056	45.802		37.047
	393.15	89.396		35.12	5.356		2.767	0.154		0.061	46.102		38.144
	373.15	104.368		42.264	6.253		3.33	0.164		0.067	49.461		42.161
	348.15	102.78		40.633	6.158		3.202	0.163		0.064	49.128		41.307
	323.15	124.855		47.597	7.481		3.751	0.176		0.067	53.348		44.738
	298.15	135.184		48.664	8.1		3.835	0.193		0.069	55.072		45.219

Table S10: Figures of merit obtained by fitting a {2,2} model (2 × Ni-O, 2 × Ni-Ni), {2,1} model (2 × Ni-O, 1 × Ni-Ni) and {1,1} model (1 × Ni-O, 1 × Ni-Ni) to the EXAFS data as a function of temperature, in the Ni-O shell and Ni-Ni shell (from 0.5 Å to 3.1 Å). $r\chi^2$ refers to reduced χ^2 and BIC refers to the Bayesian Information Criterion.[40]

S7.5 EXAFS data: Refined parameters

Here, the refined parameters from fitting the various models to the EXAFS data are presented. For the fits that were performed solely for the Ni-O shell (i.e. in the range 0.5 Å to 2.0 Å), Table S11 shows the refined parameters for the {1,0} model and Table S12 shows the refined parameters for the {2,0} model.

For the fits that were performed for the Ni-O shell and the Ni-Ni shell (i.e. in the range 0.5 Å to 3.1 Å), Table S13 shows the refined parameters for the {1,1} model. For the {2,1} and {2,2} models, the Ni-O and Ni-Ni shells are tabulated separately. Table S14 and Table S15 shows the refined parameters for the {2,1} model for the Ni-O and Ni-Ni paths respectively. Table S16 and Table S17 shows the refined parameters for the {2,2} model for the Ni-O and Ni-Ni paths respectively.

Table S11: Refined parameters for all paths from fitting a JT-undistorted {1,0} model (consisting of a single Ni-O path) to the EXAFS data for the Ni-O shell only (i.e. in the range 0.5 Å to 2.0 Å). From top to bottom are the measurements in chronological order.

		Ni-O path		
T (K)	ΔE_0 (eV)	σ^2 ($\times 10^{-3}$Å^2)	Δr (Å)	R_{eff} (Å)
298.15	-7.5 ± 1.9	8.0 ± 1.2	-0.091 ± 0.019	1.888 ± 0.019
323.15	-7.5 ± 0.2	8.1 ± 1.2	-0.091 ± 0.019	1.889 ± 0.019
348.15	-0.0 ± 700.0	8.2 ± 1.2	-0.091 ± 0.019	1.889 ± 0.019
373.15	-7.0 ± 10.0	8.5 ± 1.2	-0.089 ± 0.019	1.890 ± 0.019
398.15	-7.50 ± 0.12	8.7 ± 1.2	-0.089 ± 0.019	1.891 ± 0.019
423.15	-7.5 ± 1.3	8.9 ± 1.2	-0.087 ± 0.019	1.892 ± 0.019
443.15	-7.5 ± 1.4	9.0 ± 1.2	-0.087 ± 0.019	1.892 ± 0.019
453.15	-7.5 ± 0.5	9.1 ± 1.2	-0.087 ± 0.019	1.893 ± 0.019
463.15	-8.0 ± 5.0	9.2 ± 1.2	-0.086 ± 0.019	1.893 ± 0.019
473.15	-7.50 ± 0.04	9.3 ± 1.2	-0.085 ± 0.019	1.895 ± 0.019
483.15	-7.0 ± 3.0	9.5 ± 1.2	-0.081 ± 0.019	1.898 ± 0.019
493.15	-7.0 ± 3.0	9.6 ± 1.0	-0.055 ± 0.016	1.924 ± 0.016
503.15	-5.0 ± 3.0	9.6 ± 1.0	-0.041 ± 0.015	1.938 ± 0.015
513.15	-5.0 ± 3.0	9.6 ± 1.0	-0.041 ± 0.016	1.939 ± 0.016
523.15	-5.0 ± 3.0	9.7 ± 1.0	-0.040 ± 0.016	1.939 ± 0.016
533.15	-5.0 ± 3.0	9.8 ± 1.0	-0.038 ± 0.016	1.941 ± 0.016
513.15	-5.0 ± 3.0	9.7 ± 1.1	-0.038 ± 0.016	1.941 ± 0.016
493.15	-5.0 ± 3.0	9.5 ± 1.0	-0.039 ± 0.016	1.940 ± 0.016
473.15	-5.0 ± 3.0	9.5 ± 1.0	-0.040 ± 0.016	1.939 ± 0.016
463.15	-8.0 ± 3.0	9.6 ± 1.0	-0.068 ± 0.017	1.911 ± 0.017
453.15	-7.5 ± 0.3	9.4 ± 1.1	-0.079 ± 0.018	1.900 ± 0.018
443.15	-7.0 ± 4.0	9.2 ± 1.1	-0.082 ± 0.018	1.897 ± 0.018
433.15	-7.0 ± 2.0	9.0 ± 1.1	-0.084 ± 0.018	1.895 ± 0.018
423.15	-8.0 ± 3.0	9.0 ± 1.1	-0.084 ± 0.018	1.895 ± 0.018
413.15	-7.0 ± 3.0	8.8 ± 1.2	-0.085 ± 0.019	1.894 ± 0.019
403.15	-7.0 ± 6.0	8.7 ± 1.2	-0.086 ± 0.019	1.893 ± 0.019
393.15	-7.5 ± 1.9	8.8 ± 1.2	-0.087 ± 0.019	1.892 ± 0.019
373.15	-7.5 ± 0.3	8.5 ± 1.2	-0.087 ± 0.019	1.893 ± 0.019
348.15	-7.5 ± 0.5	8.4 ± 1.2	-0.088 ± 0.019	1.891 ± 0.019
323.15	-7.5 ± 0.2	8.3 ± 1.2	-0.088 ± 0.019	1.891 ± 0.019
298.15	-8.0 ± 3.0	8.0 ± 1.2	-0.089 ± 0.019	1.890 ± 0.019

T	ΔE_0	Ni-O path 1			Ni-O path 2		
(K)	(eV)	σ^2 ($\times 10^{-3}$Å^2)	Δr (Å)	R_{eff} (Å)	σ^2 ($\times 10^{-3}$Å^2)	Δr (Å)	R_{eff} (Å)
298.15	-5.0 ± 3.0	4.0 ± 0.6	-0.017 ± 0.012	1.896 ± 0.012	12.0 ± 6.0	0.01 ± 0.04	2.17 ± 0.04
323.15	-5.0 ± 3.0	4.1 ± 0.6	-0.017 ± 0.013	1.896 ± 0.013	13.0 ± 6.0	0.02 ± 0.04	2.17 ± 0.04
348.15	-6.0 ± 4.0	4.3 ± 0.6	-0.022 ± 0.013	1.891 ± 0.013	15.0 ± 8.0	0.03 ± 0.05	2.18 ± 0.05
373.15	-6.0 ± 4.0	4.6 ± 0.6	-0.022 ± 0.014	1.891 ± 0.014	17.0 ± 9.0	0.04 ± 0.07	2.20 ± 0.07
398.15	-7.5 ± 1.8	4.8 ± 0.6	-0.025 ± 0.014	1.888 ± 0.014	20.0 ± 12.0	0.07 ± 0.09	2.23 ± 0.09
423.15	-7.0 ± 6.0	5.0 ± 0.7	-0.024 ± 0.015	1.889 ± 0.015	22.0 ± 14.0	0.07 ± 0.11	2.23 ± 0.11
443.15	-7.50 ± 0.09	5.1 ± 0.7	-0.025 ± 0.015	1.888 ± 0.015	24.0 ± 17.0	0.08 ± 0.13	2.24 ± 0.13
453.15	-8.0 ± 5.0	5.2 ± 0.7	-0.024 ± 0.015	1.889 ± 0.015	24.0 ± 17.0	0.08 ± 0.13	2.24 ± 0.13
463.15	-7.0 ± 5.0	5.3 ± 0.7	-0.023 ± 0.015	1.890 ± 0.015	24.0 ± 18.0	0.09 ± 0.13	2.25 ± 0.13
473.15	-7.0 ± 5.0	5.4 ± 0.7	-0.022 ± 0.016	1.891 ± 0.016	30.0 ± 20.0	0.10 ± 0.15	2.26 ± 0.15
483.15	-8.0 ± 5.0	5.7 ± 0.7	-0.019 ± 0.016	1.894 ± 0.016	30.0 ± 40.0	0.1 ± 0.2	2.3 ± 0.2
493.15	-6.0 ± 14.0	6.2 ± 1.1	0.01 ± 0.17	1.92 ± 0.17	0.0 ± 200.0	-0.2 ± 1.3	1.9 ± 1.3
503.15	-4.0 ± 15.0	6.3 ± 1.2	0.0 ± 0.2	1.9 ± 0.2	0.0 ± 600.0	-0.2 ± 1.4	1.9 ± 1.4
513.15	-3.0 ± 5.0	7.0 ± 3.0	0.02 ± 0.04	1.93 ± 0.04	10.0 ± 20.0	-0.14 ± 0.18	2.02 ± 0.18
523.15	-3.0 ± 5.0	7.0 ± 3.0	0.02 ± 0.04	1.93 ± 0.04	10.0 ± 20.0	-0.14 ± 0.19	2.02 ± 0.19
533.15	-4.0 ± 16.0	6.5 ± 1.3	0.03 ± 0.13	1.94 ± 0.13	0.0 ± 200.0	-0.2 ± 1.5	1.9 ± 1.5
513.15	-4.0 ± 6.0	8.0 ± 3.0	0.02 ± 0.05	1.93 ± 0.05	10.0 ± 20.0	-0.1 ± 0.2	2.0 ± 0.2
493.15	-4.0 ± 5.0	8.0 ± 5.0	0.02 ± 0.06	1.93 ± 0.06	10.0 ± 20.0	-0.2 ± 0.2	2.0 ± 0.2
473.15	-3.0 ± 5.0	7.0 ± 3.0	0.02 ± 0.04	1.93 ± 0.04	10.0 ± 20.0	-0.14 ± 0.17	2.02 ± 0.17
463.15	-7.0 ± 11.0	6.1 ± 0.8	-0.01 ± 0.02	1.91 ± 0.02	30.0 ± 40.0	0.20 ± 0.13	2.36 ± 0.13
453.15	-7.0 ± 3.0	5.7 ± 0.7	-0.017 ± 0.017	1.896 ± 0.017	30.0 ± 50.0	0.2 ± 0.2	2.4 ± 0.2
443.15	-7.0 ± 4.0	5.5 ± 0.7	-0.020 ± 0.016	1.893 ± 0.016	30.0 ± 40.0	0.2 ± 0.2	2.3 ± 0.2
433.15	-8.0 ± 5.0	5.2 ± 0.7	-0.021 ± 0.015	1.892 ± 0.015	30.0 ± 30.0	0.13 ± 0.19	2.29 ± 0.19
423.15	-8.0 ± 5.0	5.2 ± 0.7	-0.022 ± 0.016	1.891 ± 0.016	30.0 ± 30.0	0.12 ± 0.18	2.28 ± 0.18
413.15	-8.0 ± 4.0	4.9 ± 0.7	-0.023 ± 0.015	1.890 ± 0.015	30.0 ± 20.0	0.09 ± 0.16	2.24 ± 0.16
403.15	-7.0 ± 3.0	4.8 ± 0.7	-0.023 ± 0.015	1.890 ± 0.015	23.0 ± 17.0	0.08 ± 0.13	2.24 ± 0.13
393.15	-10.0 ± 60.0	4.9 ± 0.7	-0.024 ± 0.016	1.889 ± 0.016	23.0 ± 17.0	0.08 ± 0.13	2.23 ± 0.13
373.15	-7.5 ± 1.6	4.7 ± 0.7	-0.024 ± 0.016	1.889 ± 0.016	21.0 ± 15.0	0.06 ± 0.12	2.22 ± 0.12
348.15	-7.0 ± 4.0	4.6 ± 0.7	-0.025 ± 0.015	1.888 ± 0.015	21.0 ± 14.0	0.06 ± 0.11	2.22 ± 0.11
323.15	-5.0 ± 4.0	4.3 ± 0.7	-0.018 ± 0.014	1.895 ± 0.014	15.0 ± 9.0	0.01 ± 0.06	2.17 ± 0.06
298.15	-5.0 ± 3.0	3.9 ± 0.6	-0.019 ± 0.013	1.894 ± 0.013	13.0 ± 7.0	0.00 ± 0.05	2.16 ± 0.05

Table S12: Refined parameters for all paths from fitting a JT-distorted {2,0} model (consisting of two Ni-O paths) to the EXAFS data for the Ni-O shell only (i.e. in the range 0.5 Å to 2.0 Å). From top to bottom are the measurements in chronological order.

T (K)	ΔE_0 (eV)	Ni-O path σ^2 ($\times 10^{-3}$Å^2)	Ni-O path Δr (Å)	Ni-O path R_{eff} (Å)	Ni-Ni path σ^2 ($\times 10^{-3}$Å^2)	Ni-Ni path Δr (Å)	Ni-Ni path R_{eff} (Å)
298.15	-7.5 ± 0.4	8.0 ± 1.1	-0.091 ± 0.018	1.888 ± 0.018	20.0 ± 4.0	-0.05 ± 0.04	2.91 ± 0.04
323.15	-8.0 ± 4.0	8.2 ± 1.1	-0.090 ± 0.017	1.889 ± 0.017	20.0 ± 3.0	-0.06 ± 0.03	2.90 ± 0.03
348.15	-10.0 ± 60.0	8.3 ± 1.1	-0.090 ± 0.017	1.889 ± 0.017	20.0 ± 3.0	-0.06 ± 0.03	2.90 ± 0.03
373.15	-7.0 ± 4.0	8.6 ± 1.0	-0.089 ± 0.016	1.890 ± 0.016	20.0 ± 3.0	-0.06 ± 0.03	2.90 ± 0.03
398.15	-8.0 ± 3.0	8.8 ± 1.0	-0.088 ± 0.016	1.891 ± 0.016	20.0 ± 3.0	-0.06 ± 0.03	2.90 ± 0.03
423.15	-8.0 ± 3.0	8.9 ± 1.0	-0.087 ± 0.016	1.892 ± 0.016	19.0 ± 3.0	-0.06 ± 0.03	2.90 ± 0.03
443.15	-7.50 ± 0.04	9.1 ± 1.0	-0.087 ± 0.016	1.893 ± 0.016	19.0 ± 3.0	-0.06 ± 0.03	2.90 ± 0.03
453.15	-8.0 ± 4.0	9.2 ± 1.0	-0.086 ± 0.016	1.893 ± 0.016	19.0 ± 2.0	-0.06 ± 0.03	2.90 ± 0.03
463.15	-7.5 ± 1.0	9.3 ± 1.0	-0.086 ± 0.015	1.894 ± 0.015	19.0 ± 2.0	-0.06 ± 0.03	2.90 ± 0.03
473.15	-7.5 ± 0.5	9.4 ± 1.0	-0.084 ± 0.015	1.895 ± 0.015	19.0 ± 2.0	-0.05 ± 0.03	2.91 ± 0.03
483.15	-7.5 ± 1.6	9.6 ± 1.0	-0.081 ± 0.015	1.898 ± 0.015	16.6 ± 1.6	-0.05 ± 0.02	2.91 ± 0.02
493.15	-1.7 ± 1.6	10.4 ± 1.0	-0.030 ± 0.011	1.949 ± 0.011	8.7 ± 0.5	-0.009 ± 0.01	2.951 ± 0.01
503.15	-0.9 ± 1.4	10.2 ± 0.9	-0.020 ± 0.010	1.959 ± 0.010	7.6 ± 0.4	-0.005 ± 0.008	2.955 ± 0.008
513.15	-1.1 ± 1.4	10.3 ± 1.0	-0.019 ± 0.010	1.960 ± 0.010	7.6 ± 0.4	-0.005 ± 0.008	2.956 ± 0.008
523.15	-1.0 ± 1.4	10.4 ± 1.0	-0.018 ± 0.010	1.961 ± 0.010	7.6 ± 0.4	-0.003 ± 0.008	2.957 ± 0.008
533.15	-1.0 ± 1.4	10.5 ± 0.9	-0.017 ± 0.010	1.962 ± 0.010	7.6 ± 0.4	-0.003 ± 0.008	2.957 ± 0.008
513.15	-1.3 ± 1.4	10.4 ± 1.0	-0.017 ± 0.010	1.962 ± 0.010	7.4 ± 0.4	-0.004 ± 0.008	2.956 ± 0.008
493.15	-1.2 ± 1.4	10.2 ± 1.0	-0.017 ± 0.010	1.962 ± 0.010	7.2 ± 0.4	-0.004 ± 0.008	2.956 ± 0.008
473.15	-0.7 ± 1.4	10.2 ± 1.0	-0.017 ± 0.010	1.962 ± 0.010	7.2 ± 0.4	-0.003 ± 0.008	2.957 ± 0.008
463.15	-4.0 ± 2.0	10.1 ± 1.0	-0.053 ± 0.013	1.926 ± 0.013	11.0 ± 0.7	-0.023 ± 0.013	2.937 ± 0.013
453.15	-7.0 ± 3.0	9.5 ± 1.0	-0.079 ± 0.014	1.901 ± 0.014	15.0 ± 1.3	-0.047 ± 0.019	2.913 ± 0.019
443.15	-7.0 ± 3.0	9.3 ± 1.0	-0.082 ± 0.014	1.897 ± 0.014	16.2 ± 1.6	-0.05 ± 0.02	2.91 ± 0.02
433.15	-8.0 ± 3.0	9.1 ± 1.0	-0.083 ± 0.015	1.896 ± 0.015	16.8 ± 1.8	-0.05 ± 0.02	2.91 ± 0.02
423.15	-7.5 ± 1.6	9.0 ± 1.0	-0.084 ± 0.015	1.895 ± 0.015	17.2 ± 1.9	-0.05 ± 0.02	2.91 ± 0.02
413.15	-7.5 ± 0.9	8.8 ± 1.0	-0.085 ± 0.015	1.895 ± 0.015	18.0 ± 2.0	-0.05 ± 0.03	2.91 ± 0.03
403.15	-7.5 ± 1.1	8.7 ± 1.0	-0.086 ± 0.015	1.894 ± 0.015	18.0 ± 2.0	-0.05 ± 0.03	2.91 ± 0.03
393.15	-7.5 ± 1.0	8.8 ± 1.0	-0.086 ± 0.016	1.893 ± 0.016	18.0 ± 2.0	-0.05 ± 0.03	2.91 ± 0.03
373.15	-7.0 ± 3.0	8.6 ± 1.0	-0.086 ± 0.016	1.893 ± 0.016	18.0 ± 2.0	-0.05 ± 0.03	2.91 ± 0.03
348.15	-7.0 ± 3.0	8.5 ± 1.0	-0.088 ± 0.016	1.891 ± 0.016	18.0 ± 2.0	-0.05 ± 0.03	2.91 ± 0.03
323.15	-7.5 ± 1.0	8.4 ± 1.1	-0.087 ± 0.017	1.892 ± 0.017	18.0 ± 3.0	-0.05 ± 0.03	2.91 ± 0.03
298.15	-7.50 ± 0.05	8.1 ± 1.1	-0.089 ± 0.017	1.891 ± 0.017	18.0 ± 3.0	-0.05 ± 0.03	2.91 ± 0.03

Table S13: Refined parameters for all paths from fitting a {1,1} model (consisting of a single Ni-O path and a single Ni-Ni path) to the EXAFS data for the Ni-O shell and Ni-Ni shell (i.e. in the range 0.5 Å to 3.1 Å). From top to bottom are the measurements in chronological order.

T (K)	ΔE_0 (eV)	Ni-O path 1 σ^2 ($\times 10^{-3}$Å^2)	Ni-O path 1 Δr (Å)	Ni-O path 1 R_{eff} (Å)	Ni-O path 2 σ^2 ($\times 10^{-3}$Å^2)	Ni-O path 2 Δr (Å)	Ni-O path 2 R_{eff} (Å)
503.15	-0.1 ± 1.4	6.1 ± 1.6	-0.036 ± 0.012	1.940 ± 0.012	11.0 ± 8.0	0.07 ± 0.04	2.09 ± 0.04
513.15	-0.3 ± 1.4	6.0 ± 1.6	-0.037 ± 0.012	1.939 ± 0.012	10.0 ± 7.0	0.07 ± 0.04	2.09 ± 0.04
523.15	-0.2 ± 1.4	6.0 ± 1.6	-0.036 ± 0.012	1.940 ± 0.012	9.0 ± 7.0	0.07 ± 0.04	2.09 ± 0.04
533.15	-0.2 ± 1.4	6.3 ± 1.7	-0.034 ± 0.013	1.942 ± 0.013	11.0 ± 8.0	0.07 ± 0.04	2.09 ± 0.04

Table S14: Refined parameters for all paths from fitting a {2,1} model (consisting of two Ni-O paths and a single Ni-Ni path) to the EXAFS data for the Ni-O shell and Ni-Ni shell (i.e. in the range 0.5 Å to 3.1 Å). From top to bottom are the measurements in chronological order. In this table, only the Ni-O paths are presented, with the Ni-Ni path presented in Table S15.

		Ni-Ni path		
T (K)	ΔE_0 (eV)	σ^2 ($\times 10^{-3}$Å^2)	Δr (Å)	R_{eff} (Å)
503.15	-0.1 ± 1.4	7.7 ± 0.4	-0.002 ± 0.008	2.961 ± 0.008
513.15	-0.3 ± 1.4	7.6 ± 0.4	-0.002 ± 0.008	2.961 ± 0.008
523.15	-0.2 ± 1.4	7.6 ± 0.4	-0.000 ± 0.008	2.963 ± 0.008
533.15	-0.2 ± 1.4	7.7 ± 0.4	-0.001 ± 0.008	2.962 ± 0.008

Table S15: Refined parameters for all paths from fitting a {2,1} model (consisting of two Ni-O paths and a single Ni-Ni path) to the EXAFS data for the Ni-O shell and Ni-Ni shell (i.e. in the range 0.5 Å to 3.1 Å). From top to bottom are the measurements in chronological order. In this table, only the Ni-Ni paths are presented, with the Ni-O paths presented in Table S14.

T (K)	ΔE_0 (eV)	Ni-O path 1 σ^2 ($\times 10^{-3}$Å^2)	Ni-O path 1 Δr (Å)	Ni-O path 1 R_{eff} (Å)	Ni-O path 2 σ^2 ($\times 10^{-3}$Å^2)	Ni-O path 2 Δr (Å)	Ni-O path 2 R_{eff} (Å)
298.15	-3.0 ± 2.0	3.8 ± 0.7	-0.011 ± 0.011	1.902 ± 0.011	8.0 ± 4.0	0.00 ± 0.03	2.16 ± 0.03
323.15	-3.0 ± 2.0	3.8 ± 0.6	-0.011 ± 0.010	1.902 ± 0.010	8.0 ± 4.0	0.00 ± 0.03	2.16 ± 0.03
348.15	-3.0 ± 2.0	4.0 ± 0.6	-0.012 ± 0.010	1.901 ± 0.010	9.0 ± 4.0	0.00 ± 0.03	2.16 ± 0.03
373.15	-3.0 ± 2.0	4.2 ± 0.6	-0.011 ± 0.010	1.902 ± 0.010	9.0 ± 4.0	-0.00 ± 0.03	2.16 ± 0.03
398.15	-3.0 ± 2.0	4.4 ± 0.6	-0.010 ± 0.010	1.903 ± 0.010	10.0 ± 4.0	0.00 ± 0.03	2.16 ± 0.03
423.15	-4.0 ± 2.0	4.5 ± 0.6	-0.012 ± 0.011	1.901 ± 0.011	11.0 ± 5.0	-0.00 ± 0.03	2.15 ± 0.03
443.15	-4.0 ± 2.0	4.6 ± 0.6	-0.013 ± 0.011	1.900 ± 0.011	12.0 ± 5.0	-0.01 ± 0.04	2.15 ± 0.04
453.15	-4.0 ± 2.0	4.7 ± 0.6	-0.012 ± 0.011	1.901 ± 0.011	12.0 ± 5.0	-0.01 ± 0.04	2.15 ± 0.04
463.15	-3.0 ± 2.0	4.8 ± 0.6	-0.010 ± 0.011	1.903 ± 0.011	12.0 ± 5.0	-0.00 ± 0.03	2.15 ± 0.03
473.15	-3.0 ± 2.0	4.9 ± 0.6	-0.009 ± 0.011	1.904 ± 0.011	12.0 ± 5.0	-0.01 ± 0.04	2.15 ± 0.04
483.15	-3.0 ± 2.0	5.1 ± 0.7	-0.008 ± 0.011	1.905 ± 0.011	13.0 ± 6.0	-0.02 ± 0.04	2.14 ± 0.04
493.15	-2.0 ± 2.0	5.9 ± 1.5	0.011 ± 0.014	1.924 ± 0.014	11.0 ± 8.0	-0.08 ± 0.05	2.08 ± 0.05
503.15	-1.0 ± 2.0	6.3 ± 1.9	0.021 ± 0.017	1.934 ± 0.017	11.0 ± 10.0	-0.09 ± 0.06	2.07 ± 0.06
513.15	-1.0 ± 3.0	6.1 ± 1.9	0.020 ± 0.017	1.933 ± 0.017	10.0 ± 8.0	-0.08 ± 0.05	2.08 ± 0.05
523.15	-1.0 ± 2.0	6.2 ± 2.0	0.020 ± 0.017	1.933 ± 0.017	10.0 ± 8.0	-0.08 ± 0.05	2.08 ± 0.05
533.15	-1.0 ± 3.0	7.0 ± 2.0	0.024 ± 0.018	1.937 ± 0.018	11.0 ± 10.0	-0.08 ± 0.06	2.08 ± 0.06
513.15	-1.0 ± 3.0	6.0 ± 2.0	0.022 ± 0.018	1.935 ± 0.018	10.0 ± 9.0	-0.08 ± 0.06	2.07 ± 0.06
493.15	-2.0 ± 3.0	6.0 ± 2.0	0.019 ± 0.018	1.932 ± 0.018	9.0 ± 9.0	-0.09 ± 0.05	2.07 ± 0.05
473.15	-1.0 ± 2.0	6.0 ± 1.9	0.019 ± 0.016	1.932 ± 0.016	9.0 ± 8.0	-0.08 ± 0.05	2.08 ± 0.05
463.15	-3.0 ± 2.0	5.7 ± 1.1	0.000 ± 0.012	1.913 ± 0.012	14.0 ± 9.0	-0.06 ± 0.05	2.09 ± 0.05
453.15	-4.0 ± 3.0	5.2 ± 0.8	-0.007 ± 0.012	1.906 ± 0.012	15.0 ± 8.0	-0.03 ± 0.05	2.13 ± 0.05
443.15	-3.0 ± 2.0	4.9 ± 0.7	-0.008 ± 0.011	1.905 ± 0.011	13.0 ± 6.0	-0.02 ± 0.04	2.14 ± 0.04
433.15	-3.0 ± 2.0	4.7 ± 0.7	-0.009 ± 0.011	1.904 ± 0.011	12.0 ± 6.0	-0.01 ± 0.04	2.15 ± 0.04
423.15	-3.0 ± 2.0	4.6 ± 0.7	-0.010 ± 0.011	1.903 ± 0.011	12.0 ± 6.0	-0.01 ± 0.04	2.15 ± 0.04
413.15	-3.0 ± 2.0	4.4 ± 0.6	-0.010 ± 0.011	1.903 ± 0.011	11.0 ± 5.0	-0.01 ± 0.03	2.15 ± 0.03
403.15	-3.0 ± 2.0	4.3 ± 0.6	-0.009 ± 0.010	1.904 ± 0.010	10.0 ± 5.0	-0.01 ± 0.03	2.15 ± 0.03
393.15	-3.0 ± 2.0	4.3 ± 0.6	-0.009 ± 0.011	1.904 ± 0.011	10.0 ± 4.0	-0.01 ± 0.03	2.15 ± 0.03
373.15	-4.0 ± 3.0	4.2 ± 0.7	-0.012 ± 0.011	1.901 ± 0.011	10.0 ± 5.0	-0.01 ± 0.03	2.15 ± 0.03
348.15	-3.0 ± 2.0	4.1 ± 0.7	-0.011 ± 0.011	1.902 ± 0.011	10.0 ± 5.0	-0.00 ± 0.03	2.15 ± 0.03
323.15	-4.0 ± 2.0	4.0 ± 0.7	-0.011 ± 0.011	1.902 ± 0.011	9.0 ± 4.0	-0.01 ± 0.03	2.15 ± 0.03
298.15	-4.0 ± 3.0	3.7 ± 0.7	-0.013 ± 0.011	1.900 ± 0.011	8.0 ± 4.0	-0.01 ± 0.03	2.15 ± 0.03

Table S16: Refined parameters for all paths from fitting a {2,2} model (consisting of two Ni-O paths and two Ni-Ni paths) to the EXAFS data for the Ni-O shell and Ni-Ni shell (i.e. in the range 0.5 Å to 3.1 Å). From top to bottom are the measurements in chronological order. In this table, only the Ni-O paths are presented, with the Ni-Ni paths presented in Table S17.

T	ΔE_0	Ni-Ni path 1			Ni-Ni path 2		
		σ^2	Δr	R_{eff}	σ^2	Δr	R_{eff}
(K)	(eV)	($\times 10^{-3}$Å^2)	(Å)	(Å)	($\times 10^{-3}$Å^2)	(Å)	(Å)
298.15	-3.0 ± 2.0	5.8 ± 1.6	-0.02 ± 0.02	2.83 ± 0.02	9.2 ± 1.7	-0.02 ± 0.02	3.00 ± 0.02
323.15	-3.0 ± 2.0	6.3 ± 1.7	-0.02 ± 0.02	2.83 ± 0.02	9.9 ± 1.8	-0.02 ± 0.02	3.00 ± 0.02
348.15	-3.0 ± 2.0	6.3 ± 1.7	-0.014 ± 0.02	2.831 ± 0.02	10.2 ± 1.9	-0.02 ± 0.02	3.00 ± 0.02
373.15	-3.0 ± 2.0	6.8 ± 1.8	-0.01 ± 0.02	2.83 ± 0.02	11.0 ± 2.0	-0.02 ± 0.02	3.00 ± 0.02
398.15	-3.0 ± 2.0	7.1 ± 2.0	-0.01 ± 0.02	2.84 ± 0.02	11.0 ± 2.0	-0.01 ± 0.02	3.00 ± 0.02
423.15	-4.0 ± 2.0	7.0 ± 2.0	-0.01 ± 0.02	2.84 ± 0.02	12.0 ± 2.0	-0.02 ± 0.02	3.00 ± 0.02
443.15	-4.0 ± 2.0	8.0 ± 2.0	-0.01 ± 0.02	2.84 ± 0.02	12.0 ± 3.0	-0.02 ± 0.02	3.00 ± 0.02
453.15	-4.0 ± 2.0	8.0 ± 2.0	-0.00 ± 0.02	2.84 ± 0.02	12.0 ± 3.0	-0.02 ± 0.02	3.00 ± 0.02
463.15	-3.0 ± 2.0	8.0 ± 2.0	-0.00 ± 0.02	2.84 ± 0.02	12.0 ± 3.0	-0.02 ± 0.02	3.00 ± 0.02
473.15	-3.0 ± 2.0	8.0 ± 2.0	0.00 ± 0.02	2.85 ± 0.02	12.0 ± 3.0	-0.02 ± 0.02	3.00 ± 0.02
483.15	-3.0 ± 2.0	10.0 ± 5.0	0.02 ± 0.05	2.87 ± 0.05	14.0 ± 6.0	-0.03 ± 0.04	2.99 ± 0.04
493.15	-2.0 ± 2.0	15.0 ± 11.0	0.04 ± 0.08	2.88 ± 0.08	6.2 ± 0.9	-0.059 ± 0.012	2.959 ± 0.012
503.15	-1.0 ± 2.0	15.0 ± 7.0	0.07 ± 0.1	2.91 ± 0.1	5.5 ± 0.8	-0.057 ± 0.010	2.960 ± 0.010
513.15	-1.0 ± 3.0	14.0 ± 10.0	0.07 ± 0.10	2.92 ± 0.10	5.6 ± 0.8	-0.057 ± 0.011	2.961 ± 0.011
523.15	-1.0 ± 2.0	14.0 ± 10.0	0.07 ± 0.1	2.91 ± 0.1	5.5 ± 0.8	-0.055 ± 0.011	2.962 ± 0.011
533.15	-1.0 ± 3.0	14.0 ± 9.0	0.08 ± 0.10	2.92 ± 0.10	5.7 ± 0.9	-0.055 ± 0.012	2.962 ± 0.012
513.15	-1.0 ± 3.0	14.0 ± 10.0	0.08 ± 0.11	2.92 ± 0.11	5.5 ± 0.8	-0.057 ± 0.011	2.961 ± 0.011
493.15	-2.0 ± 3.0	13.0 ± 10.0	0.06 ± 0.09	2.91 ± 0.09	5.2 ± 0.8	-0.057 ± 0.011	2.961 ± 0.011
473.15	-1.0 ± 2.0	14.0 ± 10.0	0.06 ± 0.09	2.91 ± 0.09	5.1 ± 0.8	-0.056 ± 0.010	2.962 ± 0.010
463.15	-3.0 ± 2.0	15.0 ± 3.0	0.02 ± 0.08	2.86 ± 0.08	8.1 ± 1.6	-0.062 ± 0.019	2.956 ± 0.019
453.15	-4.0 ± 3.0	11.0 ± 9.0	0.16 ± 0.08	3.01 ± 0.08	13.0 ± 7.0	-0.12 ± 0.04	2.90 ± 0.04
443.15	-3.0 ± 2.0	9.0 ± 4.0	0.01 ± 0.04	2.86 ± 0.04	12.0 ± 4.0	-0.03 ± 0.03	2.99 ± 0.03
433.15	-3.0 ± 2.0	8.0 ± 3.0	0.00 ± 0.03	2.85 ± 0.03	11.0 ± 3.0	-0.03 ± 0.02	2.99 ± 0.02
423.15	-3.0 ± 2.0	8.0 ± 3.0	0.00 ± 0.03	2.85 ± 0.03	12.0 ± 3.0	-0.02 ± 0.03	2.99 ± 0.03
413.15	-3.0 ± 2.0	8.0 ± 2.0	-0.00 ± 0.02	2.84 ± 0.02	11.0 ± 2.0	-0.02 ± 0.02	3.00 ± 0.02
403.15	-3.0 ± 2.0	8.0 ± 3.0	-0.00 ± 0.02	2.84 ± 0.02	11.0 ± 3.0	-0.02 ± 0.02	2.99 ± 0.02
393.15	-3.0 ± 2.0	7.0 ± 2.0	-0.00 ± 0.02	2.84 ± 0.02	11.0 ± 2.0	-0.02 ± 0.02	3.00 ± 0.02
373.15	-4.0 ± 3.0	7.0 ± 2.0	-0.01 ± 0.02	2.84 ± 0.02	11.0 ± 2.0	-0.02 ± 0.02	3.00 ± 0.02
348.15	-3.0 ± 2.0	7.0 ± 2.0	-0.01 ± 0.02	2.84 ± 0.02	10.0 ± 2.0	-0.02 ± 0.02	3.00 ± 0.02
323.15	-4.0 ± 2.0	6.5 ± 2.0	-0.01 ± 0.02	2.83 ± 0.02	9.5 ± 1.9	-0.02 ± 0.02	2.99 ± 0.02
298.15	-4.0 ± 3.0	5.7 ± 1.7	-0.02 ± 0.02	2.83 ± 0.02	8.6 ± 1.6	-0.03 ± 0.02	2.99 ± 0.02

Table S17: Refined parameters for all paths from fitting a {2,2} model (consisting of two Ni-O paths and two Ni-Ni paths) to the EXAFS data for the Ni-O shell and Ni-Ni shell (i.e. in the range 0.5 Å to 3.1 Å). From top to bottom are the measurements in chronological order. In this table, only the Ni-Ni paths are presented, with the Ni-O paths presented in Table S16.

S7.6 EXAFS data: Q_3 parameter

The Q_3 van Vleck[1,2] parameter is introduced in the main text and defined in Equation S4 as $Q_3 = \frac{2(l-s)}{\sqrt{3}}$ where l and s are the lengths of the long and short Ni-O bond respectively. In Table S18, this parameter is presented (using Equation S4) for all the EXAFS models and both fitted ranges. Note that here, we implicitly assume that the "long" bonds are opposite one another (i.e. separated by an angle of $\sim$180° rather than $\sim$90° via the central cation) but this cannot be known from the data.

Table S18: Tabulated $Q_3 = 2(l-s)/\sqrt{3}$ parameter obtained for each model and set of shells where fits were performed. Note that $Q_3 = 0\,\text{Å}$ for the {1,0} and {1,1} cases due to the fact that only one Ni-O path was used.

	Ni-O shell only		Ni-O and Ni-Ni shells		
T (K)	{1,0} (Å)	{2,0} (Å)	{1,1} (Å)	{2,1} (Å)	{2,2} (Å)
298.15	0	0.32 ± 0.05	0		0.30 ± 0.03
323.15	0	0.32 ± 0.05	0		0.30 ± 0.03
348.15	0	0.34 ± 0.07	0		0.30 ± 0.04
373.15	0	0.35 ± 0.08	0		0.29 ± 0.04
398.15	0	0.39 ± 0.11	0		0.30 ± 0.04
423.15	0	0.39 ± 0.12	0		0.29 ± 0.04
443.15	0	0.40 ± 0.15	0		0.29 ± 0.04
453.15	0	0.41 ± 0.15	0		0.29 ± 0.04
463.15	0	0.42 ± 0.15	0		0.29 ± 0.04
473.15	0	0.43 ± 0.17	0		0.29 ± 0.04
483.15	0	0.5 ± 0.2	0		0.27 ± 0.05
493.15	0	0.0 ± 1.5	0		0.18 ± 0.06
503.15	0	0.0 ± 1.6	0	0.18 ± 0.05	0.16 ± 0.07
513.15	0	0.1 ± 0.2	0	0.18 ± 0.04	0.16 ± 0.06
523.15	0	0.1 ± 0.2	0	0.18 ± 0.04	0.16 ± 0.06
533.15	0	0.0 ± 1.7	0	0.17 ± 0.05	0.16 ± 0.07
513.15	0	0.1 ± 0.2	0		0.16 ± 0.07
493.15	0	0.1 ± 0.2	0		0.16 ± 0.06
473.15	0	0.1 ± 0.2	0		0.17 ± 0.06
463.15	0	0.52 ± 0.15	0		0.21 ± 0.06
453.15	0	0.5 ± 0.2	0		0.26 ± 0.06
443.15	0	0.5 ± 0.2	0		0.27 ± 0.05
433.15	0	0.5 ± 0.2	0		0.28 ± 0.05
423.15	0	0.4 ± 0.2	0		0.28 ± 0.05
413.15	0	0.41 ± 0.18	0		0.28 ± 0.04
403.15	0	0.40 ± 0.15	0		0.29 ± 0.04
393.15	0	0.40 ± 0.15	0		0.29 ± 0.04
373.15	0	0.38 ± 0.14	0		0.29 ± 0.04
348.15	0	0.38 ± 0.12	0		0.29 ± 0.04
323.15	0	0.32 ± 0.07	0		0.29 ± 0.04
298.15	0	0.31 ± 0.06	0		0.29 ± 0.04

References

(1) Van Vleck, J. H. The Jahn–Teller Effect and Crystalline Stark Splitting for Clusters of the Form XY_6. *The Journal of Chemical Physics* **1939**, *7*, 72–84.

(2) Nagle-Cocco, L. A. V.; Dutton, S. E. Van Vleck Analysis of Angularly Distorted Octahedra using VanVleckCalculator. *IUCr Journal of Applied Crystallography* **2024**, *57*.

(3) Kanamori, J. Crystal distortion in magnetic compounds. *Journal of Applied Physics* **1960**, *31*, S14–S23.

(4) Nagle-Cocco, L. A. V. VanVleckCalculator (GitHub): https://github.com/lnaglecocco/VanVleckCalculator (accessed 20th August 2024). 2023; `https://github.com/lnaglecocco/VanVleckCalculator`.

(5) Goodenough, J. B.; Wold, A.; Arnott, R. J.; Menyuk, N. J. P. R. Relationship between crystal symmetry and magnetic properties of ionic compounds containing Mn^{3+}. *Physical Review* **1961**, *124*, 373.

(6) Rodriguez-Carvajal, J.; Hennion, M.; Moussa, F.; Moudden, A. H.; Pinsard, L.; Revcolevschi, A. J. Neutron-diffraction study of the Jahn–Teller transition in stoichiometric $LaMnO_3$. *Physical Review B* **1998**, *57*, R3189.

(7) Capone, M.; Feinberg, D.; Grilli, M. Stabilization of A-type layered antiferromagnetic phase in $LaMnO_3$ by cooperative Jahn–Teller deformations. *The European Physical Journal B-Condensed Matter and Complex Systems* **2000**, *17*, 103–109.

(8) Chatterji, T.; Fauth, F.; Ouladdiaf, B.; Mandal, P.; Ghosh, B. Volume collapse in $LaMnO_3$ caused by an orbital order-disorder transition. *Physical Review B* **2003**, *68*, 052406.

(9) Zhou, J.-S.; Goodenough, J. B. Orbital mixing and ferromagnetism in $LaMn_{1-x}Ga_xO_3$. *Physical Review B* **2008**, *77*, 172409.

(10) Zhou, J.-S.; Alonso, J.; Han, J.; Fernández-Díaz, M.; Cheng, J.-G.; Goodenough, J. B. Jahn–Teller distortion in perovskite $KCuF_3$ under high pressure. *Journal of Fluorine Chemistry* **2011**, *132*, 1117–1121.

(11) Snamina, M.; Oleś, A. M. Spin-orbital order in the undoped manganite $LaMnO_3$ at finite temperature. *Physical Review B* **2016**, *94*, 214426.

(12) Fedorova, N. S. et al. Relationship between crystal structure and multiferroic orders in orthorhombic perovskite manganites. *Physical Review Materials* **2018**, *2*, 104414.

(13) Lindner, F. P.; Aichhorn, M.; Banerjee, H. Interplay of dynamic correlations and uniaxial strain driving magnetic phase transitions in $LaMnO_3$. *arXiv preprint arXiv:2212.01090* **2022**,

(14) Alonso, J. A.; Martinez-Lope, M. J.; Casais, M. T.; Fernández-Díaz, M. T. Evolution of the Jahn–Teller distortion of MnO_6 octahedra in $RMnO_3$ perovskites (R= Pr, Nd, Dy, Tb, Ho, Er, Y): a neutron diffraction study. *Inorganic Chemistry* **2000**, *39*, 917–923.

(15) Wang, J.; Wang, Z. D.; Zhang, W.; Xing, D. Y. Intermediate spin state stabilized by the Jahn–Teller distortion in $La_{1/2}Ba_{1/2}CoO_3$. *Physical Review B* **2002**, *66*, 064406.

(16) Tachibana, M.; Shimoyama, T.; Kawaji, H.; Atake, T.; Takayama-Muromachi, E. Jahn–Teller distortion and magnetic transitions in perovskite $RMnO_3$ (R= Ho, Er, Tm, Yb, and Lu). *Physical Review B* **2007**, *75*, 144425.

(17) Zhou, J.-S.; Goodenough, J. B. Intrinsic structural distortion in orthorhombic perovskite oxides. *Physical Review B* **2008**, *77*, 132104.

(18) Castillo-Martínez, E.; Bieringer, M.; Shafi, S. P.; Cranswick, L. M. D.; Alario-Franco, M. Á. Highly Stable Cooperative Distortion in a Weak Jahn–Teller d^2 Cation: Perovskite-Type $ScVO_3$ Obtained by High-Pressure and High-Temperature Transformation from Bixbyite. *Journal of the American Chemical Society* **2011**, *133*, 8552–8563.

(19) Franchini, C.; Archer, T.; He, J.; Chen, X.-Q.; Filippetti, A.; Sanvito, S. Exceptionally strong magnetism in the 4d perovskites $RTcO_3$ (R= Ca, Sr, Ba). *Physical Review B* **2011**, *83*, 220402.

(20) Chiang, F.-K.; Chu, M.-W.; Chou, F. C.; Jeng, H. T.; Sheu, H. S.; Chen, F. R.; Chen, C. H. Effect of Jahn–Teller distortion on magnetic ordering in Dy(Fe, Mn)O_3 perovskites. *Physical Review B* **2011**, *83*, 245105.

(21) Dong, S.; Zhang, Q.; Yunoki, S.; Liu, J.-M.; Dagotto, E. Magnetic and orbital order in $(RMnO_3)_n/(AMnO_3)_{2n}$ superlattices studied via a double-exchange model with strain. *Physical Review B* **2012**, *86*, 205121.

(22) Fedorova, N. S.; Ederer, C.; Spaldin, N. A.; Scaramucci, A. Biquadratic and ring exchange interactions in orthorhombic perovskite manganites. *Physical Review B* **2015**, *91*, 165122.

(23) Ji, C.; Wang, Y.; Guo, B.; Shen, X.; Luo, Q.; Wang, J.; Meng, X.; Zhang, J.; Lu, X.; Zhu, J. Strain engineering of magnetic and orbital order in perovskite $LuMnO_3$ epitaxial films. *Physical Review B* **2019**, *100*, 174417.

(24) Xu, L.; Meng, J.; Liu, Q.; Meng, J.; Liu, X.; Zhang, H. Strategy for achieving multiferroic E-type magnetic order in orthorhombic manganites $RMnO_3$ (R= La-Lu). *Physical Chemistry Chemical Physics* **2020**, *22*, 4905–4915.

(25) Ren, W.-N.; Jin, K.; Guo, E.-J.; Ge, C.; Wang, C.; Xu, X.; Yao, H.; Jiang, L.; Yang, G. Strain-engineered high-temperature ferromagnetic oxygen-substituted $NaMnF_3$ from first principles. *Physical Review B* **2021**, *104*, 174428.

(26) Moron, M. C.; Palacio, F.; Rodríguez-Carvajal, J. Crystal and magnetic structures of $RbMnF_4$ and $KMnF_4$ investigated by neutron powder diffraction: the relationship between structure and magnetic properties in the Mn^{3+} layered perovskites $AMnF_4$ (A= Na, K, Rb, Cs). *Journal of Physics: Condensed Matter* **1993**, *5*, 4909.

(27) Cussen, E. J.; Rosseinsky, M. J.; Battle, P. D.; Burley, J. C.; Spring, L. E.; Vente, J. F.; Blundell, S. J.; Coldea, A. I.; Singleton, J. Control of Magnetic Ordering by Jahn–Teller Distortions in Nd_2GaMnO_6 and La_2GaMnO_6. *Journal of the American Chemical Society* **2001**, *123*, 1111–1122.

(28) Wang, J.; Zhang, W.; Xing, D. Y. Role of Jahn–Teller effect in the charge ordered states of $YBaM_2O_5$ (M= Co, Mn). *Physical Review B* **2002**, *66*, 052410.

(29) Delmas, C.; Saadoune, I.; Dordor, P. Effect of cobalt substitution on the Jahn–Teller distortion of the $NaNiO_2$ layered oxide. *Molecular Crystals and Liquid Crystals Science and Technology. Section A. Molecular Crystals and Liquid Crystals* **1994**, *244*, 337–342.

(30) Tummers, B. 2006; https://datathief.org/.

(31) Neuefeind, J.; Feygenson, M.; Carruth, J.; Hoffmann, R.; Chipley, K. K. The nanoscale ordered materials diffractometer NOMAD at the spallation neutron source SNS. *Nuclear Instruments and Methods in Physics Research Section B: Beam Interactions with Materials and Atoms* **2012**, *287*, 68–75.

(32) Momma, K.; Izumi, F. VESTA 3 for three-dimensional visualization of crystal, volumetric and morphology data. *Journal of Applied Crystallography* **2011**, *44*, 1272–1276.

(33) Van Rossum, G.; Drake, F. L. *Python 3 Reference Manual*; CreateSpace: Scotts Valley, CA, 2009.

(34) Ong, S. P.; Richards, W. D.; Jain, A.; Hautier, G.; Kocher, M.; Cholia, S.; Gunter, D.; Chevrier, V. L.; Persson, K. A.; Ceder, G. Python Materials Genomics (pymatgen): A robust, open-source python library for materials analysis. *Computational Materials Science* **2013**, *68*, 314–319.

(35) Ahmed, M. R.; Gehring, G. A. Potts model for the distortion transition in $LaMnO_3$. *Physical Review B* **2006**, *74*, 014420.

(36) Chung, J.-H.; Proffen, T.; Shamoto, S.; Ghorayeb, A. M.; Croguennec, L.; Tian, W.; Sales, B. C.; Jin, R.; Mandrus, D.; Egami, T. Local structure of $LiNiO_2$ studied by neutron diffraction. *Physical Review B* **2005**, *71*, 064410.

(37) Baur, W. H. The geometry of polyhedral distortions. Predictive relationships for the phosphate group. *Acta Crystallographica Section B: Structural Crystallography and Crystal Chemistry* **1974**, *30*, 1195–1215.

(38) Coelho, A. A. TOPAS and TOPAS-Academic: an optimization program integrating computer algebra and crystallographic objects written in C++. *Journal of Applied Crystallography* **2018**, *51*, 210–218.

(39) Norberg, S. T.; Tucker, M. G.; Hull, S. Bond valence sum: a new soft chemical constraint for RMCProfile. *Journal of Applied Crystallography* **2009**, *42*, 179–184.

(40) Neath, A. A.; Cavanaugh, J. E. The Bayesian information criterion: background, derivation, and applications. *Wiley Interdisciplinary Reviews: Computational Statistics* **2012**, *4*, 199–203.

(41) Radin, M. D.; Van der Ven, A. Simulating charge, spin, and orbital ordering: application to Jahn–Teller distortions in layered transition-metal oxides. *Chemistry of Materials* **2018**, *30*, 607–618.

(42) Chung, J. S.; Thorpe, M. F. Local atomic structure of semiconductor alloys using pair distribution functions. *Physical Review B* **1997**, *55*, 1545.
